# Unveiling pathogens and contaminants: refining metagenomics for clinical diagnostics

**DOI:** 10.3389/fmicb.2026.1786985

**Published:** 2026-04-02

**Authors:** Marta Ibañez-Lligoña, Sergi Colomer-Castell, Carolina Campos, Álvaro González-Camuesco, Arnau Llauradó, Jorgina Garcia-Larroy, Daniel Sánchez-Tejerina, Ariadna Rando-Segura, Cristina Andrés, Juliana Esperalba, Patricia Nadal, Roser Ferrer, Maria Francesca Cortese, David Tabernero, Josep Gregori, Mar Riveiro-Barciela, Juan Carlos Ruiz-Cobo, Agustin Ruiz, Ester del Barco, Maria Buti, Maria Goya, Andrés Antón, Amanda Cano, Raúl Juntas-Morales, Josep Quer

**Affiliations:** 1 Liver Diseases-Viral Hepatitis, Liver Unit, Vall d’Hebron Institut de Recerca (VHIR), Instituto de Investigación Sanitaria Hospital Universitari Vall d’Hebron (IIS IR-HUVH), Barcelona, Spain; 2 Medicine Department, Universitat Autònoma de Barcelona (UAB), Bellaterra, Spain; 3 Centro de Investigación Biomédica en Red de Enfermedades Hepáticas y Digestivas (CIBERehd), Madrid, Spain; 4 Biochemistry and Molecular Biology Department, UAB, Bellaterra, Spain; 5 Neuromuscular Diseases Unit, Neurology Department, IIS IR-HUVH, Vall d’Hebron Barcelona Hospital Campus, Barcelona, Spain; 6 Peripheral Nervous System Group, VHIR - IIS IR-HUVH, Vall d’Hebron Barcelona Hospital Campus, Barcelona, Spain; 7 European Reference Network on Rare Neuromuscular Diseases (ERN EURO-NMD), Barcelona, Spain; 8 Microbiology Department, VHIR - IIS IR-HUVH, Vall d’Hebron Barcelona Hospital Campus, Barcelona, Spain; 9 Centro de Investigación Biomédica en Red de Enfermedades Infecciosas (CIBERINFEC), Madrid, Spain; 10 Biochemistry Department, Vall d'Hebron Hospital Universitari, Vall d'Hebron Barcelona Hospital Campus, Barcelona, Spain; 11 Research Center and Memory Clinic, ACE Alzheimer Center Barcelona, Universitat Internacional de Catalunya, Barcelona, Spain; 12 Ciber Neurodegenerative Diseases (CIBERNED), Madrid, Spain; 13 Department of Microbiology, Immunology and Molecular Genetics, Long School of Medicine, Glenn Biggs Institute for Alzheimer's & Neurodegenerative Diseases, The University of Texas Health Science Center, San Antonio, TX, United States; 14 Maternal-Fetal Medicine Unit, Department of Obstetrics, VHIR - IIS IR-HUVH, Vall d’Hebron Barcelona Hospital Campus, Barcelona, Spain; 15 Department of Pediatrics, Obstetrics and Gyneacology, and Preventive Medicine and Public Health, Universitat Autònoma de Barcelona (Bellaterra), Bellaterra, Spain

**Keywords:** coinfection, diagnostics, metagenomics, sequencing, virology

## Abstract

**Introduction:**

Shotgun metagenomic sequencing (mNGS), an untargeted approach that sequences all nucleic acids in a sample, has emerged as a powerful tool for pathogen detection and genome characterization. However, its implementation in clinical diagnostics remains limited due to technical challenges such as contamination and reduces sensitivity, especially in low-biomass samples.

**Methods:**

We applied mNGS to 144 clinical samples representing chronic infections, acute infections, and respiratory co-infections. To address contamination, we established a framework integrating negative controls, lab-specific contaminant watchlists, and computational filtering. Viral detection performance and genome recovery were assessed across sample types and viral loads.

**Results:**

Viral load was shown to be the primary determinant of sensitivity, with reliable recovery achieved only at higher titers. Our framework substantially improved contamination management, reducing false-positive signals and enhancing viral genome recovery. mNGS enabled the detection of clinically relevant co-infections and refined viral classification beyond targeted diagnostics, while also revealing the substantial risk of spurious detections in the absence of contamination-aware workflows.

**Discussion:**

These findings define practical sensitivity thresholds for clinical mNGS and underscore the need for contamination-aware workflows, particularly for low-biomass samples, while providing an open-source contaminants watchlist that enhances reliability and utility of clinical metagenomics.

## 1 Introduction

Shotgun metagenomics (mNGS) is a next-generation sequencing (NGS) approach that enables comprehensive analysis of all nucleic acids (both DNA and RNA), within a given sample (Quer et al., 2022; Ibañez-Lligoña et al., 2023). By using a non-targeted strategy, mNGS allows for unbiased sequencing of microbial and host genetic material, facilitating the detection and characterization of a wide range of microorganisms, including viruses, bacteria, fungi, archaea, and parasites (Chrzastek et al., 2022; Parras-Moltó et al., 2018; Iyer and Damania, 2020; Xie et al., 2023; Zhao et al., 2024; Miao et al., 2018; Su et al., 2024). This hypothesis-free approach is particularly well suited for identifying novel or unexpected pathogens and understanding microbial diversity and dynamics in complex clinical settings (Ibañez-Lligoña et al., 2023). Metagenomics has proven to be valuable in a wide range of applications including study of microbial community composition, pathogen evolution, antimicrobial resistance, and the discovery of novel microorganisms through whole-genome sequencing (Quer et al., 2022; Chiu and Miller, 2019; von Fricken et al., 2023).

Over the last decade, mNGS has been introduced into the clinical sphere (Comas et al., 2020), where it offers several advantages over conventional diagnostics (Chiu and Miller, 2019; Gu et al., 2019). Traditional methods such as culture, PCR or serological tests rely on prior knowledge of the target organism and fail to identify unculturable, unknown, or unexpected pathogens (Ibañez-Lligoña et al., 2023; Schloss and Handelsman, 2005). Moreover, whole-genome sequencing enables functional characterization, identification of resistance mutations and virulence factors, thereby supporting both diagnosis and informed therapeutic decision-making.

Accurate disease diagnosis is essential for effective treatment, as misdiagnoses causes delays, increased mortality and higher healthcare costs. Acute infections remain particularly challenging, with 50–60% of hospitalized patients being discharged without an identified cause. In emerging infections and chronic conditions, low-abundance or resolved infections often go undetected by conventional methods. Since syndromic presentations can result from multiple pathogens, a high-throughput approach that simultaneously identifies all potential agents in one assay is needed (Edridge et al., 2019). Moreover, mNGS could be used for prompt detection of emerging, re-emerging, or novel pathogens is essential for timely outbreak containment (Quer et al., 2022).

Despite its potential, clinical implementation of mNGS remains limited due to technical, analytical and interpretative challenges (Ibañez-Lligoña et al., 2023). Contamination is one of the major technical barriers, which is exacerbated by the technique’s high sensitivity (Jurasz et al., 2021). Exogenous nucleic acids can be introduced at multiple stages of the workflow, from sample collection to sequencing (Fierer et al., 2025). Common sources include reagents and consumables (collectively known as the” kitome” [Olomu et al., 2020)], laboratory environments, and operator handling (Lou et al., 2023). These contaminants can lead to false-positive results or obscure genuine microbial signals. This is particularly critical in low-biomass samples, where the actual amount of microbial genetic material is very small compared to the host or background content, therefore enabling contaminants and host-derived reads dominate the sequencing output (Jurasz et al., 2021; Fierer et al., 2025; Minich et al., 2019).

Several strategies have been developed to mitigate the risk of contamination, including physical decontamination methods (e.g., UV treatment, DNase digestion), bioinformatic filtering, and the use of negative controls (Fierer et al., 2025; Corless et al., 2000). However, no single approach has proven to fully remove background noise. In addition, few studies have systematically catalogued the composition and behaviour of contaminants across large clinical datasets or assessed their impact on diagnostic performance. This knowledge gap complicates efforts to set detection thresholds or confidently interpret results from low-biomass samples. In this study, we aim to address key limitations in clinical mNGS workflows by characterizing recurrent contamination and evaluating the natural viral sensitivity across 144 clinical samples and 18 methodological controls.

## 2 Materials and methods

### 2.1 Sample collection

A total of 144 clinical samples were collected from patients across various diagnostic groups representing diverse diagnostic categories and subjected to shotgun metagenomic sequencing (Table 1). All samples were leftover material obtained from routine clinical diagnostic testing. Samples were completely anonymized. Human data of the participants related to sex, gender, race, ethnicity, social grouping, and other social variables are not relevant to the scope of the analysis. Our research has been approved by the Vall d’Hebron Barcelona Hospital Campus Ethics Committee under the following approval numbers: PR(AG)429–2021 and PR(AG)287–2022 for neuropathy samples, PR(AG)259–2020 for the respiratory virus samples, PR(AG)118–2021 for HEV samples, and PR(AMI)437–2023 for pregnant women samples. Written informed consent was obtained from all patients for the collection and research use of their biological samples.

**Table 1:** Distribution of the 144 samples analyzed, categorized by sample type and indicating paired samples.

Condition	Sample type	Article code	Number of samples	Paired samples
Alzheimer’s disease	CSF	ALZ-CSF	39	NA
Guillain-Barré Syndrome	CSF	GBS-CSF	26	21
	Serum	GBS-S	21	
Neuralgic amyotrophy	CSF	NA-CSF	1	1
	Serum	NA-S	1	
Preterm birth	Plasma	PB-P	4	4
	Amniotic fluid	PB-AF	6	
Positive controls	Nasopharyngeal swab	PC-NFS	11	NA
	Nasopharyngeal exudate	PC-NFE	4	
	Plasma	PC-P	10	
Acute HEV	Amniotic fluid	HEVA-AF	1	2
	Feces	HEVA-F	1	
	Plasma	HEVA-P	2	
Chronic HEV	Plasma	HEVC-P	17	NA

The codification depending on the condition and type is added for easy interpretation of the results. AF, amniotic fluid; ALZ, Alzheimer’s disease; CSF, cerebrospinal fluid; F, feces; GBS, Guillain-Barré Syndrome; HEV, Hepatitis E virus; HEVA, Acute Hepatitis E virus infection; HEVC, Chronic Hepatitis E virus infection, P, plasma; PB, preterm birth; PC, positive control; NA, neuralgic amyotrophy; NFE, Nasopharyngeal exudate; NFS, nasopharyngeal swab.

Among these, samples from patients with Guillain-Barré Syndrome (GBS) were also analyzed, which included 26 cerebrospinal fluid (CSF) and 21 serum specimens, of which 21 of them form matched CSF-serum pairs. Both serum and CSF samples were collected in the context of acute symptomatology for suspected GBS cases, with posterior diagnostic confirmation of included cases. Additionally, 1 matched CSF-serum pair was obtained from a case of neuralgic amyotrophy, another acute inflammatory peripheral nervous system disorder. CSF was obtained through a lumbar puncture performed for clinical diagnostic purposes, with a surplus of 2 to 4 mL retained for research. The CSF samples were then frozen at −80 °C for preservation. For serum collection, whole blood was drawn into 9 mL blood tubes and centrifuged at 3500 rpm at 4 °C for 15 min. The resulting serum was aliquoted into 1.5 mL tubes. Serum was then frozen at −80 °C for preservation.

Additionally, 39 CSF samples were collected from individuals diagnosed with Alzheimer’s disease (AD), following the methodology described in the Cano et al. (2024). Six amniotic fluid samples and four plasma samples, including four matched pairs, were collected from pregnant women in the clinical context of suspected intra-amniotic infection (chorioamnionitis). Gestational ages at sampling ranged from 16 to 33.6 weeks. Upon hospital admission, an amniocentesis was performed under ultrasound guidance for diagnostic purposes. Using continuous ultrasound monitoring, a sterile needle was inserted via transabdominal puncture to aspirate a sample of amniotic fluid (AF) under strict aseptic conditions.

In addition to the mentioned clinical samples, a set of positive controls was included consisting of clinical specimens with confirmed viral infections using conventional clinical microbiology methods. Eleven nasopharyngeal swabs, four nasopharyngeal exudates and ten plasma samples were obtained from cases of suspected acute infection in patients admitted to the hospital or emergency department. Moreover, one stool, one amniotic fluid and two plasma samples were collected from a patient which had tested positive for a HEV acute infection. Furthermore, 17 plasma samples were obtained from a patient with a chronic HEV infection.

Samples were classified as low-biomass or high-biomass according to their anatomical origin. Low-biomass samples were defined as specimens derived from physiologically sterile body sites, including cerebrospinal fluid, plasma, serum, and amniotic fluid, which are not expected to harbour a resident microbiome under basal conditions. High-biomass samples comprised specimen types known to contain abundant microbial communities, such as stool and nasopharyngeal swabs and exudates (Fierer et al., 2025). Blank negative controls consisted of DNAse and RNase-free sterile water (ThermoFisher Scientific, Waltham, MA, USA), which is routinely used for nucleic acid extractions and RT-PCR-Nested reactions.

### 2.2 Nucleic acid extraction, library preparation and Illumina sequencing

For nucleic acid extraction from nasopharyngeal swab and exudates, the STARMag 96 × 4 Universal Cartridge Kit was used on the Microlab STARled automated platform (Seegene, South Korea). In contrast, RNA and DNA from amniotic fluid, cerebrospinal fluid, plasma, serum and stool samples were extracted using the QIAamp MinElute Virus Spin extraction kit (QIAGEN, Hilden, Germany), omitting the addition of the RNA carrier. Next, sequencing libraries were prepared through the TruSeq Stranded total RNA library kit (Illumina, San Diego, CA, USA) according to the manufacturer’s instructions. Unique dual indices (IDT for Illumina – TruSeq RNA UD Indexes v2) were ligated to each sample to enable multiplexing. Following library quantification and normalization to 4 nM, libraries were pooled prior to sequencing.

Additionally, two types of negative methodological controls were included in the workflow: extraction controls (CE), added during nucleic acid extraction (sterile water), and library controls (CL), added during library preparation and processed in parallel with the clinical samples throughout the workflow. A total of eighteen “blank” controls were included across different sequencing runs to assess and control for inter and intra-run sequencing variations.

Library quality was assessed using the KAPA Library Quantification Kit (Roche Applied Science, Pleasanton, CA, USA) by RT-qPCR in the Light Cycler 480 instrument in combination with the TapeStation 4,200 system (Agilent, Santa Clara, CA, USA) using the D1000 ScreenTape Assay. Finally, libraries were sequenced on either the NextSeq2000 or Novaseq6000 platforms (Illumina, San Diego, CA, USA), with PhiX v3 (Illumina, San Diego, CA, USA) used as internal control for sequencing.

Sequencing generated a total of 9.81 × 10^10^ reads across all eleven runs. Two of these runs were performed in a NovaSeq6000 platform, while the other nine runs were done in the NextSeq2000 platform. Libraries sequenced on the NextSeq2000 platform produced a median of 9.53 × 10^7^ reads per sample (3.4 × 10^6^–1.54 × 10^8^), whereas NovaSeq6000 runs yielded a median of 1.61 × 10^8^ reads per sample (6.67 × 10^7^–4.51 × 10^8^ reads).

### 2.3 Cross-contamination control experiment

Additionally, three experiments were carried out to assess the putative cross-contamination that occurred during the process of library preparation and/or sequencing. For that, a NextSeq2000 (Illumina, San Diego, CA, USA) was loaded with a pool containing eight water “blank” controls, a previously produced in-lab HCV clone and one RNA coming from HEVC-P-2 sample which underwent library preparation through TruSeq Stranded total RNA library kit (Illumina, San Diego, CA, USA).

### 2.4 Bioinformatic analysis

Sequencing data was retrieved from the sequencing platforms in either FASTQ or BCL format for all clinical samples and corresponding negative controls. When required, BCL files were converted to FASTQ format using the bcl2fastq software (Illumina, San Diego, CA, USA). Then, samples underwent an initial quality control (QC) assessment.

QC analysis began with the generation of quality reports using FastQC (Babraham Bioinformatics, 2010) and MultiQC (Ewels et al., 2016). Next, raw reads were trimmed using Trimmomatic (Bolger et al., 2014) to remove adapter sequences, low-quality bases, and short reads. Duplicate sequences were removed using the clumpify tool from BBMap suite (Bushnell, 2014). To minimize the influence of technical contamination, BBDuk from BBMAP suite (Bushnell, 2014) was used to filter out reads present in matched extraction (CE) and library (CL) controls.

Human reads were identified by mapping against the human reference genome (GRCh38) using Bowtie2 (Langmead and Salzberg, 2012) and subsequently removed from the dataset. The remaining non-human reads were subjected to taxonomic classification using Kraken2 (Wood et al., 2019) with the PlusPF database (RefSeq database for archaea, bacteria, viruses, plasmids, human, UniVec_Core, protozoa and fungi; accessed in April 2025). Taxonomic results were then filtered using an in-house script to remove known environmental and reagent-derived contaminants, based on both internal negative controls and previously published contaminant lists. *De novo* assembly of high-quality, non-human reads was performed using MEGAHIT (Li et al., 2015). Assembly quality was evaluated by remapping reads back to the assembled contigs. Assembled contigs were further classified taxonomically using Kraken2 (Wood et al., 2019). To validate the presence of specific pathogens identified through taxonomic classification and to assess genome coverage, reference-based mapping was conducted on selected positive samples using Bowtie2 (Langmead and Salzberg, 2012).

All downstream analyses were conducted in R (R Core Team, 2022) (v 4.2.5). Data processing and visualization, including Principal Coordinate Analysis, bar plots, line graphs, and statistical summaries, were performed using the tidyverse (Wickham et al., 2019), ggplot2 (Wickham, 2016), and other relevant R packages. Custom scripts were used to generate read-retention plots across pipeline steps, exploring taxonomic profiles and compare microbial compositions between sample groups and control types. In parallel, decontam (Davis et al., 2018) was used to identify contaminants in our data.

To contextualize the presence of recurrent contaminant genera in clinical samples, the proportion of non-human reads assigned to genera included in the defined contaminant watchlist was calculated for each sample using raw reads counts prior to normalization. Moreover, rarefaction (saturation) analyses were performed in host-depleted, pre-filtered genus-level files to assess sequence depth adequacy across sample types with the use of the R package vegan (Oksanen et al., 2025).

## 3 Results

### 3.1 Characterization of recurrent contaminants in negative controls

Negative controls showed highly consistent contaminant profiles dominated by recurrent taxa, with no significant differences between extraction vs. library controls. Notably, the next step involving host read depletion altered overall compositional clustering patterns and reduced the total read counts, indicating that high host content dominates Bray–Curtis dissimilarity when present and can obscure microbial-specific variation (Supplementary Figures S1, S2).

To assess variability in community composition across control types, we performed PERMANOVA and beta-dispersion analyses after each processing step. No significant differences were detected between extraction and library controls at any stage (PERMANOVA *p*-value > 0.05, dispersion p-value > 0.05). Although statistical testing did not reveal any significant difference in beta dispersion at the human-read-removed stage, there is an apparent difference in spread between extraction and library controls observed in the dispersion boxplot (Supplementary Figure S3).

After filtering, a contaminant watchlist was generated by identifying taxonomic families (Supplementary Table S1) and genera (1271) (Supplementary Table S2) that were prevalent in at least half of all negative controls and had a counts per million (CPM) > 0.5 (Table 2). From this list, 81 genera such as *Propionibacterium*, *Gammaretrovirus* or *Flavobacterium*, matched previously published contaminants (Jurasz et al., 2021; Lou et al., 2023; Asplund et al., 2019; Piro and Renard, 2022; Zinter et al., 2019; Salter et al., 2014; Hornung et al., 2019; Miller et al., 2019) (Supplementary Table S3). Moreover, we extracted specific genera according to methodological control type (Supplementary Tables S4, S5).

**Table 2:** Top 15 most prevalent and abundant genera detected across all methodological controls.

Genus	NCBI taxonomy ID	Prevalence (%)	Mean CPM	Median CPM	Interquartile range of CPM
*Phyllobacterium*	28100	100.00	434929.23	746526.62	770279.24
*Burkholderia*	32008	100.00	166363.15	7837.61	360049.24
*Escherichia*	561	100.00	40490.29	4817.07	34218.42
*Alcaligenes*	507	100.00	18907.37	774.93	39799.55
*Pseudomonas*	286	100.00	16802.39	3018.45	4813.42
*Micrococcus*	1269	100.00	13736.17	1327.76	6938.39
*Cutibacterium*	1912216	100.00	11400.18	3053.41	22247.96
*Sphingomonas*	13687	100.00	10633.93	1176.37	3583.25
*Moraxella*	475	100.00	10615.59	671.47	9709.87
*Homo*	9605	100.00	9350.71	4009.78	4866.26
*Mesorhizobium*	68287	100.00	9274.51	5730.84	15776.96
*Corynebacterium*	1716	100.00	7134.13	1714.06	8339.69
*Shewanella*	22	100.00	6549.39	424.87	10470.74
*Paraburkholderia*	1822464	100.00	5699.98	670.65	3090.06

The table includes genus name, NCBI Taxonomy ID, and abundance statistics: mean counts per million (CPM), calculated from raw genus-level read counts and normalized to the total number of reads per sample. Summary statistics (mean, median, and interquartile range (IQR)) are shown across all negative controls.

Application of this contaminant watchlist reduced the total number of genera to be assessed for downstream analysis in clinical samples by 44.7%, substantially simplifying clinical reporting. After host removal, the proportion of non-host reads classified as recurrent contaminants from the watchlist varied across sample types. Among low-biomass samples, amniotic fluid showed the highest mean percentage of contaminant reads (98.6%), followed by serum (97.7%), cerebrospinal fluid (96.9%), and plasma (94.4%).

High-biomass samples showed greater variability. Nasopharyngeal exudates (97.3%), nasopharyngeal swabs (92.6%) displayed contaminant proportions comparable to low-biomass samples, whereas the fecal sample showed a substantially lower proportion of contaminant reads (48.9%) consistent with a higher endogenous microbial content (Supplementary Tables S6, S7).

To explore sample-control relationships, PCoA was performed on 151 samples (including replicates) and 18 blank negative controls after each pipeline step. Samples were stratified by biomass level (low-biomass samples, high-biomass samples). Moreover, rarefaction (saturation) analyses were performed stratified by clinical sample type to evaluate sequencing depth adequacy (Supplementary Figures S4–S6). Across sample types and methodological controls, curves demonstrated early plateauing behaviour indicating that sequencing depth was sufficient to capture the majority of the detectable taxa within each matrix.

**Figure 1:**
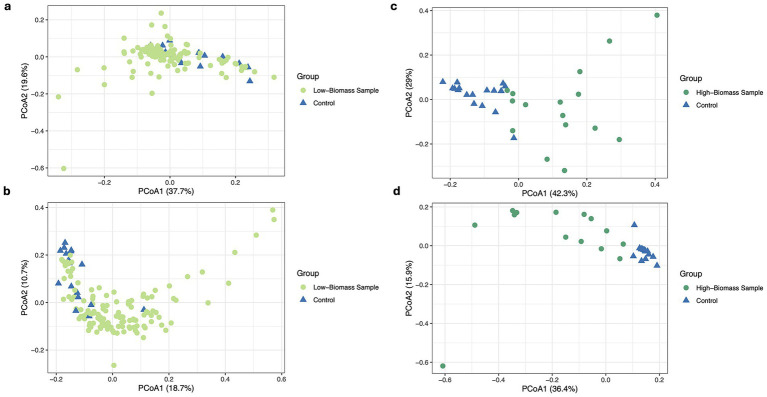
Principal coordinates analysis (PCoA) at genus level based on Bray-Curtis distances, showing clinical samples and methodological controls. **(a)** Low-biomass samples after initial preprocessing (adapter and quality trimming). **(b)** Low-biomass samples after BBDuk (Bushnell, 2014) read subtraction decontamination strategy. **(c)** High-biomass samples after initial preprocessing (adapter and quality trimming). **(d)** High-biomass samples after BBDuk (Bushnell, 2014) read subtraction decontamination.

In early processing steps (after the removal of low-quality sequences), low-biomass samples clustered tightly with controls, and PERMANOVA revealed no significant compositional differences (PERMANOVA *p*-value > 0.05, dispersion *p*-value > 0.05, Figure 1a), indicating the domination of contamination, due to limited true microbial signal. For decontamination, we employed BBDuk (Bushnell, 2014)-based read subtraction using negative controls as reference. This led to partial separation of low-biomass samples from controls in the Bray-Curtis space (Figure 1b), although the PCoA still showed overlapping. PERMANOVA confirmed a statistically significant difference between low-biomass samples and methodological negative controls (R^2^ = 0.053, *p*-value < 0.001). However, beta dispersion analysis indicated a significantly higher within-group variability (*F* = 25.0, *p*-value < 0.001), suggesting that heterogeneity in dispersion may partially contribute to the observed group differences. Conversely, PCoA of high-biomass samples and controls showed a clear separation from controls as early as the trimming step (PERMANOVA *p*-value < 0.05) (Figure 1c). This trend continued across the subsequent pipeline stages, and by the last step, separation was pronounced, with PCoA1 explaining 36.4% of the variance (Figure 1d). In contrast, low-biomass samples clustered tightly with controls across steps.

To further reduce background noise, we applied prevalence-based filtering using decontam (Davis et al., 2018), alongside our contaminant watchlist for interpretative purposes to flag ambiguous or low abundance taxa without risking the exclusion of true biological signals (Supplementary Figure S7). Despite elevated group heterogeneity (beta dispersion *p*-value < 0.05), the total retained microbial abundance per sample was significantly higher in clinical samples compared to negative control (Wilcoxon *p*-value < 0.001), supporting the presence of true biological content distinct from background contamination.

### 3.2 Assessment of cross-contamination and index hopping

**Figure 2:**
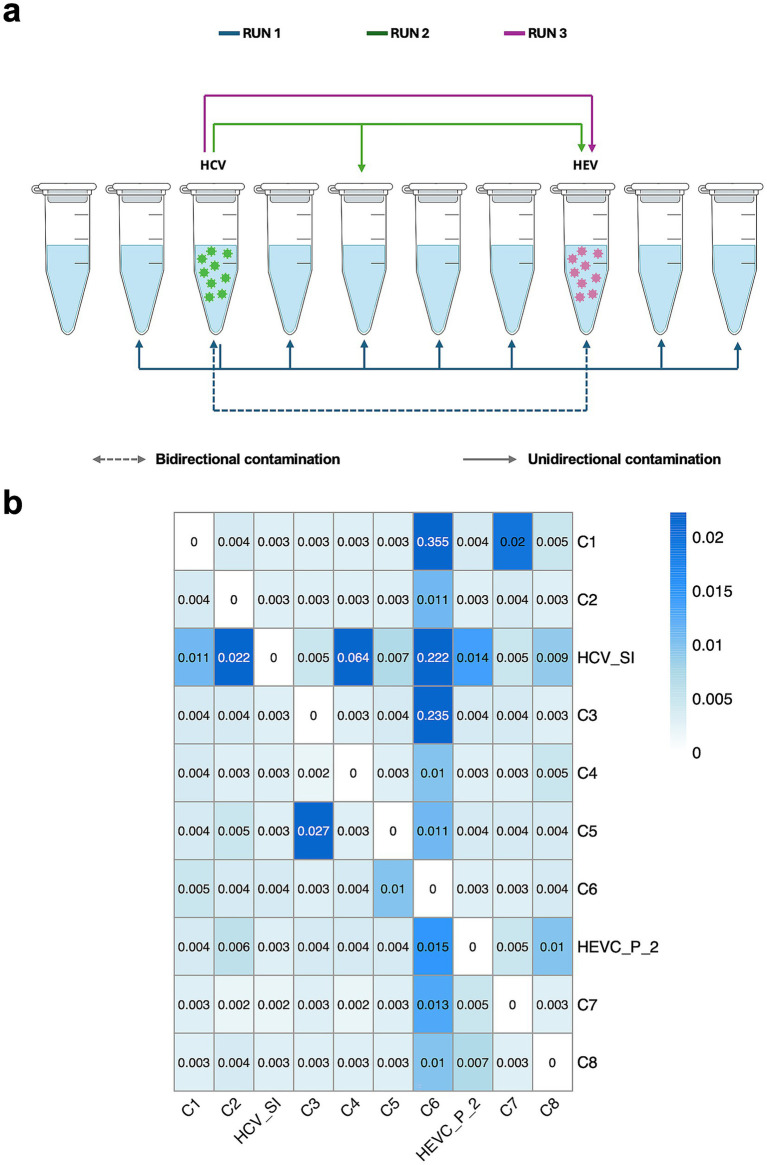
Cross-contamination results. **(a)** Schematic figure of the three cross-contamination experiments when performing library preparation. Arrows indicate the presence of viral reads detected in each sample post-sequencing and post-upstream analysis, demonstrating cross-contamination. Green virions in the third tube represents the presence of HCV in the tube, while purple virions in the eight tube represents the presence of HEV. **(b)** Heatmap showing the mean percentage of reads from the three sequencing runs misassigned from donor samples (rows) to receiver samples (columns), indicative of index hopping during Illumina sequencing. The values represent the percentage of reads erroneously assigned across sample indexes.

To evaluate cross-contamination, nine negative controls (sterile water), one of which was spiked with a hepatitis C Virus (HCV) clone were sequenced. A hepatitis E virus (HEV)-positive plasma sample was included in the same batch in three separate sequencing runs. Despite stringent protocols, HCV reads were detected in other samples in all three sequencing runs at significantly lower read counts (between 2 to 161 reads) compared to the HCV sample. HEV reads appeared only in the HEV samples in two out of the three replicate sequencing runs. However, HEV reads appeared in the HCV-spiked sample at very low read counts in one of the batches (Figure 2a).

To further investigate these low-level detections, we analyzed shared tile and index combinations across samples to identify potential index misassignment events in the three sequencing runs. This analysis revealed pairwise misassignment events across samples in the three sequencing runs, with a pattern of dual-index sharing consistent with index hopping. Most sample pairs showed minimal read transfer (<0.01%), indicating low background cross-contamination. However, a few pairs displayed higher misassignment rates (0.2–0.5%), consistent within the previously reported range for index hopping (0.1–2%) (https://sapac.illumina.com/techniques/sequencing/ngs-library-prep/multiplexing/index-hopping.html) (Figure 2b).

More specifically, the HCV-spiked sample, which exhibited a strong abundance gradient, low-level HCV reads were detected in other samples, consistent with potential transfer from high-abundance libraries. However, index-sharing analysis does not allow unequivocal determination of read origin, and directionality is inferred based on relative abundance patterns rather than directly observed.

### 3.3 mNGS diagnostic performance across clinical infection types

#### 3.3.1 Assessment of a chronic infection

**Figure 3:**
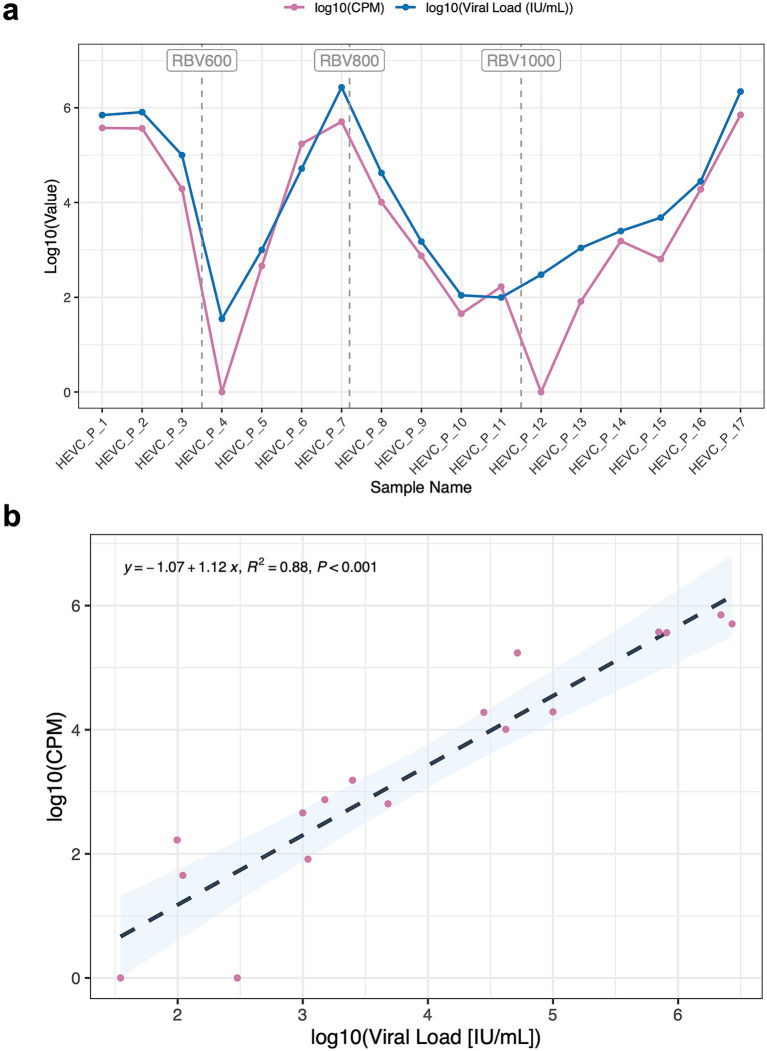
Viral load vs metagenomic detection (CPM). **(a)** Line plot showing log-transformed viral load (IU/mL) for hepatitis E virus (HEV) and its corresponding log-transformed viral counts per million. **(b)** Correlation plot between log-transformed viral load and log-transformed normalized read counts (CPM).

We analyzed 17 longitudinal plasma samples from a patient with a chronic HEV infection, viral loads ranged from 35 IU/mL to x 2.2 10^6^ IU/mL. Metagenomic sequencing along with background subtraction, successfully detected HEV reads in 15 out of 17 samples. Detection was consistent at viral loads above 10^3^ IU/mL, with HEV reads ranging from 82.09 to 7.10 × 10^5^ counts per million (CPM) (Supplementary Table S8). In contrast, samples with viral loads lower than 10^3^ IU/mL showed inconsistent or no detection of HEV (Figure 3), despite testing qPCR-positive cases. This suggests that 10^3^ IU/mL is the lowest viral load at which detection becomes reproducible and retrieves reliable detection by mNGS. In addition, we observed a strong positive correlation between the log-transformed viral load (IU/mL) and the log-transformed normalized read counts (CPM) obtained by metagenomic sequencing (Pearson’s r = 0.93, *p*-value < 0.001), indicating that higher viral loads lead to a proportionally enhanced detection of the pathogen. This correlation underscores the quantitative potential of metagenomic sequencing in virome studies and highlights detection limits relevant to clinical diagnostics.

In samples with viral loads above 5 × 10^4^ IU/mL, near-complete recovery was achieved, with > 99% genome coverage, CPM values exceeding 170,000, and full-length assemblies generated as single contigs (N50 = longest contig = total length ≈ 7,200 bp) (Supplementary Table S8; Supplementary Figure S8). In contrast, samples with low viral loads (< 10^3^ IU/mL), which exhibited inconsistent HEV detection, showed minimal or no genome coverage, CPMs below 1,000, and failed to yield contig assemblies, underscoring the reduced sensitivity of metagenomic sequencing at low input concentrations. Intermediate viral load cases (e.g., HEVC-P-8, HEVC-P-9, HEVC-P-16) produced partial genome coverage (24–75%) and fragmented assemblies composed of multiple shorter contigs with reduced N50 values, suggesting a gradual relationship between input viral load and assembly completeness (Supplementary Table S8).

#### 3.3.2 Acute viral infections

We analyzed 14 clinical samples from patients with serologically and RT-qPCR confirmed acute viral infections. These included ten plasma samples (low-biomass) with Cytomegalovirus (CMV), Human polyomavirus 1 (BKV) and Epstein–Barr virus (EBV), with viral loads ranging from 226 IU/mL to 2.87 × 10^5^ IU/mL. In addition, four samples from a patient with acute HEV genotype 1 infection were analyzed: two plasma samples, one amniotic fluid sample (all low biomass), and one fecal sample (high biomass).

mNGS detected its qPCR-confirmed pathogen in eleven out of fourteen samples (Table 3). Consistent with our prior observations, detection became unreliable below viral loads of 10^3^ IU/mL. Moreover, the log-transformed viral load (IU/mL) strongly correlated with log-transformed CPM values (Pearson’s r = 0.77, *p*-value < 0.05), further confirming that higher viral loads correlate with higher read counts in metagenomics, reinforcing the threshold for reliable viral detection in both chronic and acute infection contexts (Supplementary Figure S9). Viral load also correlated positively with genome coverage (Pearson’s r = 0.61, p-value < 0.05), with coverage spanning from 0% in low-titer samples to up to 100% in two HEV samples (HEVA-P-1, HEVA-F-1). While viral load was the main driver of detection and assembly, some exceptions (PC-P-3) suggest that factors such as read quality, library preparation biases, or strain divergence also influence assembly and detection success.

**Table 3:** Summary of metagenomic sequencing and viral assembly metrics for positive clinical samples with acute infections from Epstein–Barr virus (EBV), cytomegalovirus (CMV) human polyomavirus 1 (BKV), and hepatitis E virus (HEV).

Sample ID	Target virus	Viral load (IU/mL)	CPM	Genome covered (%)	Number of contigs	N50	Longest contig	Total length
PC-P-1	CMV	23,208	13083.15	11.07	6	386	996	2,571
PC-P-2	EBV	25,372	296044.21	74.93	101	667	3,256	63,292
PC-P-3	EBV	2,868,857	2070.93	1.37	NA	NA	NA	NA
PC-P-4	CMV	30,592	2578.88	4.76	1	362	362	362
PC-P-5	CMV	226	43.17	0.08	NA	NA	NA	NA
PC-P-6	EBV	1,430,851	23575.64	16.81	3	996	996	1761
PC-P-7	BKV	4,215	249.07	4.48	NA	NA	NA	NA
PC-P-8	EBV	1,690	0.00	0.00	NA	NA	NA	NA
PC-P-9	EBV	17,832	0.56	0.30	NA	NA	NA	NA
PC-P-10	EBV	4,550	49.65	0.38	NA	NA	NA	NA
HEVA-P-1	HEV	1.01×10^8^	163754.15	100	1	7,192	7,192	7,192
HEVA-F-1	HEV	2.1×10^10^	421568.63	100	1	7,278	7,278	7,278
HEVA-P-2	HEV	120	0.00	0.00	0	NA	NA	NA
HEVA-AF-2	HEV	44	0.00	0.00	0	NA	NA	NA

The table includes Sample ID, target virus identified via qPCR in clinical diagnostics, viral load (IU/mL), counts per million (CPM) of the corresponding virus, percentage of the viral genome covered by reads, number of assembled contigs classified as the target virus, N50,the length of the longest contig, and the length of all viral contigs. NA = not applicable, due to absence of sufficient reads for contig assembly.

Despite successful taxonomic detection, only six of the 14 samples yielded contigs corresponding to the expected pathogen (Table 3; Supplementary Figures S10–S13). Contig recovery ranged from a single 362 bp CMV contig in a plasma sample (viral load of 30,592 IU/mL, sample PC-P-4) to a 7,278 bp HEV contig in the fecal sample HEVA-F-1. Interestingly, additional viral sequences unrelated to the primary infection were identified. In sample PC-P-9, we recovered four contigs attributed to Human betaherpesvirus 6A (N50 = 509 bp, longest contig = 567 bp, total length = 1,940 bp) and three contigs corresponding to GB Virus C in the same sample (N50 = 7,767, longest contig = 7,767 bp, total length = 10,020). These findings underscore the potential of mNGS to detect coinfections or latent viral elements beyond the targeted pathogen.

#### 3.3.3 Viral coinfections in respiratory samples

**Figure 4:**
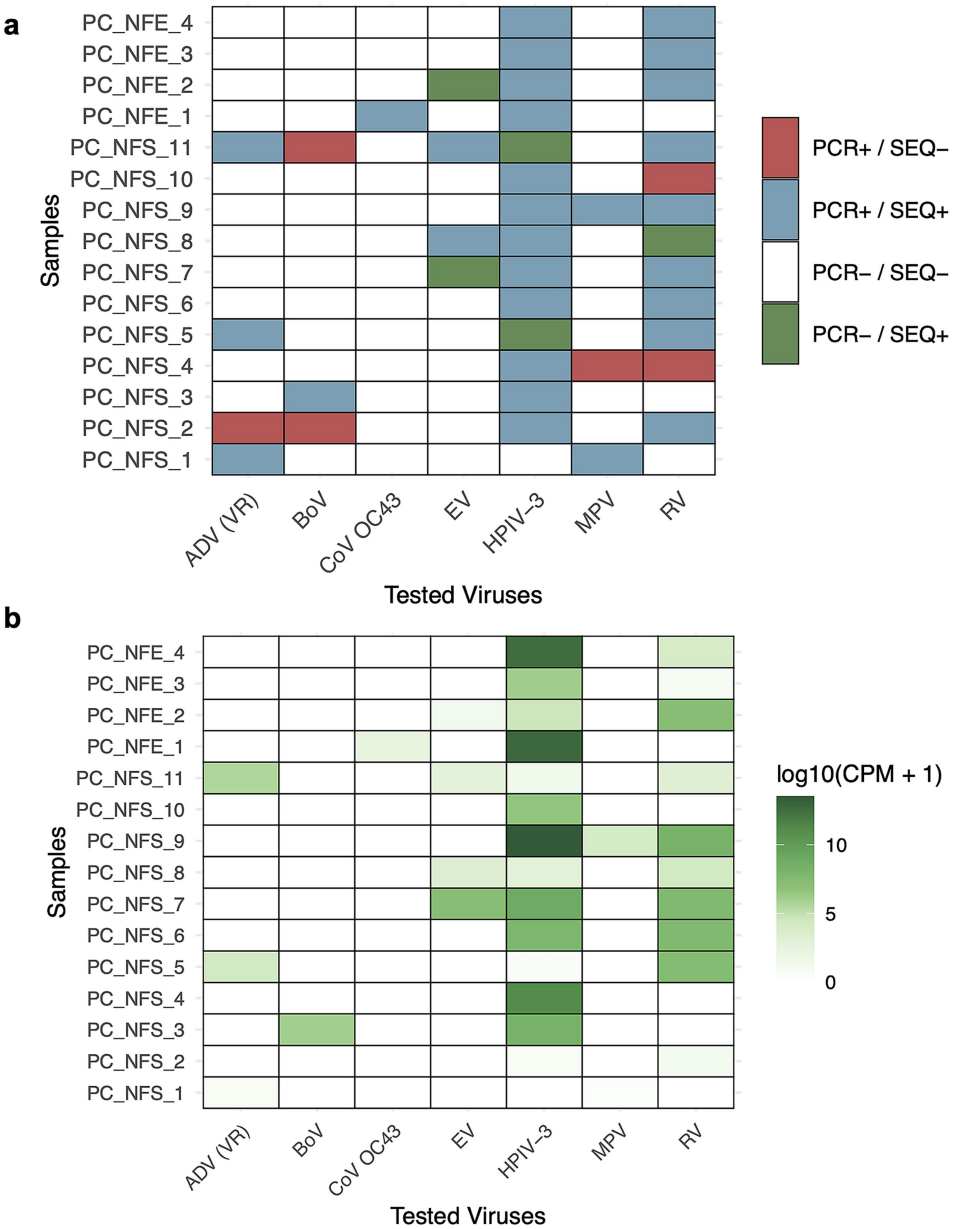
Detection of respiratory viruses in coinfections. **(a)** Heatmap summarizing the detection status of tested viruses across all samples using qPCR and sequencing. Each tile represents a sample-virus pair and can be found color-coded by detection outcome. **(b)** Heatmap showing log-transformed viral read counts (CPM) of virus-specific sequencing reads across samples. CPM was calculated as the number of virus-specific sequencing reads normalized to the total number of non-host reads per sample. Each tile represents a sample-virus pair and is color-coded by normalized viral sequencing abundance. The tested viruses included adenovirus [ADV (VR)], bocavirus (BoV), human coronavirus OC43 (CoV OC43), enterovirus (EV), human respirovirus 3 (HPIV-3), human metapneumovirus (MPV), and rhinovirus (RV).

To evaluate mNGS performance in detecting viral coinfections, we analyzed 15 nasopharyngeal swabs and exudates previously tested by RT-qPCR for multiple respiratory viruses, including adenovirus (AdV), bocavirus (BoV), Human coronavirus OC43 (CoV OC43), Human respirovirus 3 (HPIV-3), enterovirus (EV), Human metapneumovirus (MPV), and rhinovirus (RV). The rationale for using respiratory samples to study multiple infections is that the respiratory tract is constantly exposed to environmental microorganisms. Each sample carried coinfections involving two to four viruses, with Ct values spanning from 16.15 to 36.73 for HPIV-3 (Supplementary Table S9). Detection was broadly consistent with RT-qPCR results, showing 84% concordance. Notably, HPIV-3 remained detectable through the technique at Ct values as high as 36.7, suggesting strong sensitivity for this virus. In contrast, ADV and BoV were not consistently above Ct 30. Samples with Ct values < 25 produced high read counts, while detection above Ct > 33 was variable and seemed virus dependent. Additionally, EV, RV and HPIV-3 were identified in samples that had tested negative for these through RT-qPCR (Figure 4). Beyond confirming known coinfections, mNGS revealed additional viral taxa not targeted by routine diagnostics, as seen in the clinical sample PC-P-9. These included Epstein–Barr virus (PC-NFE-3; CPM = 10.08), human betaherpesvirus 6B (PC-NFE-1, CPM = 3.11; PC-NFS-11, CPM = 47.29), human mastadenovirus B, parechovirus A1 (PC-NFS-3, CPM = 4.39), human coronavirus 229E (PC-NFS-2, CPM = 0.91) and influenza C virus (PC-NFS-8, CPM = 12.52), among others detected at very low CPM levels. Notably, sample PC_NFE_1, initially tested positive for CoV OC43 using a commercial multiplex RT-qPCR assay, while mNGS yielded a near-complete genome of human coronavirus HKU1 (longest contig = 22.3 kb).

This suggests possible cross-reactivity or limitations in strain-level resolution in targeted diagnostics and highlights the added value of mNGS for refining or correcting viral classification. Near-complete genomes were recovered for HPIV-3 and RV when RT-qPCR Ct values were < 25, with partial assemblies still achievable up to Ct ≈ 28. At higher Ct, genome recovery was inconsistent, likely reflecting reduced input material (Supplementary Table S9). Some exceptions were observed for small viral genomes (e.g., BoV and RV), which could be assembled even at moderate Ct values. Conversely, fragmented assemblies were obtained for some viruses with low Ct values, such as AdV and EV, likely due to genome complexity, low coverage uniformity, or library preparation bias.

## 4 Discussion

Our study implemented a comprehensive metagenomic framework to assess contamination control and evaluate the intrinsic sensitivity of shotgun sequencing in real clinical samples. The consistent detection of contaminant genera such as Propionibacterium and Gammaretrovirus across negative controls aligns with previous reports (Gu et al., 2019; Jurasz et al., 2021; Lou et al., 2023; Zinter et al., 2019; Salter et al., 2014) on the “kitome,” reinforcing the need for rigorous contamination filtering to mitigate their dominating effect, specifically in low-biomass samples, where these can dominate over genuine clinical signals (Zinter et al., 2019).

These findings support the feasibility of building cross-laboratory contaminant reference sets to complement local filtering. Importantly, this contaminant watchlist is taxon-based: the recurrent detection of reads assigned to a given genus in negative controls does not necessarily imply that the entire organism is intrinsically a contaminant, but rather that low-level signals from this taxon are consistently observed across runs. The source of contaminant sequences in metagenomic datasets often remains unclear, potentially arising from reagents, laboratory environments, sample collection and personnel handling during extraction and library preparation, emphasizing the need for standardized contamination management practices. This could be done by implementing rigorous negative controls, developing lab-specific contaminant watchlists, and incorporating computational subtraction strategies (Hornung et al., 2019; Porter et al., 2021), such as decontam (Davis et al., 2018) or metathresholds (https://github.com/sarah-buddle/metathresholds) to distinguish true biological signals from background noise. However, distinguishing true signals from contamination remains particularly challenging for taxa that are biologically plausible in the sample context (e.g., skin or respiratory-associated microbes), underscoring the need to combine wet- and dry-lab decontamination strategies. Therefore, the contaminant watchlist we have generated is not intented as an exclusion list, but as an interpretative framework to contextualize genera identified in samples. Clinical interpretation should integrate both abundance metrics, coverage distribution, and clinical context before attributing pathogenic relevance.

Moreover, we acknowledge that the specific composition of the contaminant watchlist is inherently dependent on the local laboratory environment (extraction kits, library preparation methods, operational workflows…). Therefore, the exact taxa identified in this study may not be universally generalizable across institutions, although some contaminants have already been reported in the literature, as stated in the results.

Our bioinformatics approach, which combined BBDuk (Bushnell, 2014) read subtraction with prevalence-based filtering using decontam (Davis et al., 2018), enhanced community differentiation beyond what either method achieved alone, while increasing within-group dispersion. This likely arises from the compounded effects of aggressive contaminant filtering and biological heterogeneity in low-biomass samples. This is not necessarily undesirable, it may signify genuine biological heterogeneity, consistent with known principles of microbiome variation in low-biomass or stressed environments (Davis et al., 2018; Austin and Korem, 2024). Although the analytical workflow used in this study is based on previously described bioinformatic approaches, our objective was to evaluate its performance and practical applicability in real-world clinical samples, including plasma and respiratory specimens with varying viral loads. Our results provide an integrated assessment of its ability to detect and characterize viral sequences in challenging low-biomass samples, highlighting both its diagnostic potential and technical limitations. This type of systematic evaluation in clinical specimens is essential to support the implementation of metagenomic sequencing in routine diagnostic settings, where factors such as host background, sequencing depth, and analytical filtering can significantly influence performance.

In high-biomass samples, contaminants contribute less compared to true biological content, reducing the impact on downstream analyses. As a result, such samples may not require the same level of decontamination stringency as low-biomass samples although contamination-aware workflows and negative controls remain essential (Jurasz et al., 2021). Moreover, persistent contamination underscores a major clinical risk, potentially causing false positives, inappropriate treatment, or missed infections.

While environmental and reagent-based contamination has been widely studied cross-sample contamination remains comparatively unexplored (Jurasz et al., 2021; Lou et al., 2023; Zinter et al., 2019; Salter et al., 2014). To address this, we conducted a controlled sequencing run using spiked and unspiked negative controls. Despite stringent procedures, read carryover was observed, showing that cross-sample contamination can compromise interpretation even under controlled conditions, especially in low-biomass samples. This highlights the need for careful experimental designs that include multiple types of negative controls (Fierer et al., 2025) (sampling, extraction and library). Dual-index misassignment further illustrates how technical artifacts can mimic coinfections or suggest false pathogens. Internal spike-in controls may help establish run-specific positivity thresholds but replicating this contamination assay across additional independent sequencing runs are needed to confirm reproducibility. Future advances in library preparation, barcoding, and QC metrics could further reduce index bleed-through and enhance mNGS reliability.

In viral diagnostics, metagenomic sequencing offers an untargeted approach for pathogen detection and genome characterization (Gu et al., 2019). However, its sensitivity remains a key limitation compared to targeted molecular assays such as qPCR (Ibañez-Lligoña et al., 2023), being highly dependent on input viral load. To establish clinically actionable sensitivity benchmarks, we analyzed samples from three representative infection contexts: chronic infections, acute infections, and respiratory co-infections, ensuring thresholds are directly applicable to real-world diagnostic practices. For the chronic infection context, we analyzed plasma samples from a patient with chronic HEV infection undergoing ribavirin treatment. Ribavirin, a mutagenic nucleoside analog, complicates genome recovery as it increases intra-host diversity and the burden of low-frequency mutations (Colomer-Castell et al., 2023). These changes hinder consensus accuracy and full-genome assembly, which complicates genome characterization when relying on specific primers, and for which metagenomics could provide an advantage. Our findings show that metagenomic sequencing can detect and recover HEV genomes in chronic infections, with reliable detection at 10^3^ IU/mL and nearly complete genome recovery only achievable when viral loads exceeded ~ 5 × 10^4^ IU/mL. Thus, despite its value for unbiased pathogen discovery, metagenomics cannot replace qPCR for pathogen detection yet and integrating viral load data remains essential for interpreting results.

Extending the analysis to acute infections, we applied mNGS to thirteen low-biomass plasma samples and one high-biomass fecal sample from patients with acute infections. Despite the challenges of working with low-biomass samples, metagenomics detected the target virus in eleven out of the thirteen plasma samples, demonstrating its potential utility for untargeted diagnostics. As observed in the HEV chronic infection series, detection strongly correlated with viral load and samples with viral loads below 10^3^ IU/mL failed to yield detectable metagenomic evidence of infection, reinforcing the existence of a practical sensitivity threshold for this approach (Buddle et al., 2024). Contig-level identification was only achieved in six of the samples, highlighting the importance read-level classification.

Near-complete HEV genomes were recovered from both acute infection samples with high viral loads (~ 10^8^–10^10^ IU/mL; HEVA-P-1 and HEVA-F-1), illustrating how elevated viral titers substantially enhance genome recovery. It is important to note that HEV’s small genome (~7.2 kb) likely facilitates its amplification and assembly in metagenomic workflows (Nayfach et al., 2020; Smits et al., 2015; Smits et al., 2014). Furthermore, the presence of HEV in plasma as intact viral particles contributes to deep and uniform coverage, improving the chances of assembling complete or near-complete genomes (Shahini et al., 2024; Manso et al., 2017).

In contrast, CMV, EBV and BKV are predominantly present in plasma as fragmented cell-free DNA (Parikh and Anderson, 2025; Peddu et al., 2020; Lam et al., 2018), making full-genome assembly more challenging, yet metagenomics is able to detect them (Sam et al., 2021). Importantly, this approach also revealed coinfections, reinforcing the power of mNGS as a diagnosis tool and highlighting its potential to uncover infections often missed by conventional diagnostics. Metagenomic sequencing was further evaluated in nasopharyngeal swabs and exudate samples from patients with RT-qPCR-confirmed coinfections involving two to four respiratory viruses. Metagenomic detection was broadly concordant with RT-qPCR.

However, while molecular diagnostics offer high sensitivity and specificity, they are inherently limited by their targeted nature and may miss unexpected or emerging viruses. Moreover, its implementation in routine clinical practice remains constrained by substantial financial costs, the need for specialized laboratory infrastructure, and advanced bioinformatic expertise. In addition, standardized analytical pipelines, quality control frameworks and regulatory accreditation for clinical interpretation are not yet universally established. In contrast, metagenomics is capable of simultaneously detecting known pathogens, uncovering additional viral agents not included in diagnostic panels, and even resolving strain-level identities. For instance, the recovery of a near-complete genome of human coronavirus HKU1 in a sample initially diagnosed as CoV OC43 highlights the added value of mNGS in refining viral classification and detecting closely related species.

Nevertheless, some viruses such as ADV and EV, were more difficult to assemble, possibly due to greater genome complexity or uneven read distribution. Beyond confirming known infections, mNGS also detected additional viruses such as EBV or human herpesvirus 6B, in addition to viruses which had tested negative through RT-qPCR (HPIV-3, RV and EV). These findings highlight metagenomics’ ability to uncover unexpected coinfections, raising questions about their clinical relevance, especially in immunocompromised hosts or in cases where latent or reactivating viruses may contribute to disease. Moreover, we were able to recover almost full genomes for HPIV-3, RV and human coronavirus HKU1, possibly due to smaller genome sizes (Nayfach et al., 2020; Smits et al., 2015; Smits et al., 2014) and the nature of NP swabs, which typically exhibit lower levels of host nucleic acid background (Wylie et al., 2012), thereby improving signal-to-noise ratios.

Despite the effectiveness of our combined decontamination strategy, distinguishing true low-abundance signals from residual contamination remains challenging. Including negative controls that match each sample could improve baselines and interpretation of borderline detections. Additionally, while Kraken2 (Wood et al., 2019) provides rapid and computationally efficient taxonomic classification, its reliance on k-mers can introduce misclassifications due to conserved sequences which may be shared across phylogenetically diverse organisms. Consequently, low-level taxonomic assignments to unexpected environmental taxa may arise. Such signals may therefore reflect systematic classification background inherent to database structure and k-mer matching strategies, rather than true biological presence. Importantly, our objective was not to infer the biological origin of these taxa, but to identify recurrent background assignments observed in negative controls and across sample types. Future studies may benefit from combining multiple classifiers or applying alignment-based confirmation to improve accuracy and taxonomic resolution.

The key contribution for this study is a pragmatic framework for establishing and applying a contamination watchlist in clinical shotgun metagenomic workflows, particularly for low-biomass samples. The proposed approach consists of: (1) generating multiple methodological negative controls, including both extraction and library controls processed alongside clinical samples in multiple sequencing runs; (2) sequencing these controls using the same laboratory and bioinformatic pipeline; (3) identifying taxa that are recurrently detected across controls; and (4) flagging these taxa as potential contaminants candidates during interpretation of clinical datasets. Rather than attempting to eliminate all background taxa, this framework aims to contextualize detections to reduce overinterpretation. Moreover, methodological controls should be added in all forthcoming sequencing runs as contamination monitoring is dynamic due to batch-to-batch variations in reagents, laboratory conditions, and handling practices that can introduce new contaminants over time. We do not propose a fixed number of controls as universally sufficient. Rather, laboratories should generate enough controls to allow recurrent background taxa to stabilize. The number required will depend on workflow complexity, sample types to be analyzed, and reagent variability.

The benefit of this approach is interpretative clarity: by pre-characterizing recurrent background taxa, laboratories can substantially reduce the number of genera requiring manual clinical review and focus attention on biologically plausible, sample-consistent organisms, which provides a structured interpretative layer to improve reporting consistency and transparency in clinical metagenomic diagnostics.

In summary, our study systematically evaluated contamination in viral metagenomics, highlighting the necessity of rigorous decontamination workflows, particularly in low-biomass samples frequently used in clinical settings. By integrating methodological controls, computational filtering, and lab-specific contaminant watchlists, we present a reproducible framework that enhances data interpretation and genome recovery in clinical metagenomics. Importantly, our analyses across different infections establish context-specific benchmarks, enabling laboratories to interpret metagenomics results with greater confidence. These findings underscore the continued need for contamination-aware pipelines and standardized controls in clinical metagenomics and suggest that, while mNGS holds substantial promise for broad-spectrum pathogen detection, its accuracy hinges on robust experimental design and data curation. Moving forward, the integration of quantitative measures (e.g., viral load), improved decontamination tools, and cross-laboratory contaminant reference sets will be critical to enhance diagnostic reliability and biological insight.

## Data Availability

The datasets generated and analyzed in this study are publicly available in the NCBI SRA repository (Sequence Read Archive) under the BioProject accession number PRJNA1424541. All scripts used for the data analysis will be made available upon request by contacting the corresponding author via email.
