# Supplementary material for: Unveiling pathogens and contaminants: refining metagenomics for clinical diagnostics
Source: Front Microbiol. 2026 Apr 2;17:1786985. doi: 10.3389/fmicb.2026.1786985 (PMC13083213; doi:10.3389/fmicb.2026.1786985)
Supplement: Supplementary file 5 [file Presentation_1.pdf]

Supplementary Material for "Unveiling pathogens and contaminants: refining metagenomics for clinical diagnostics"

1 Supplementary Figures

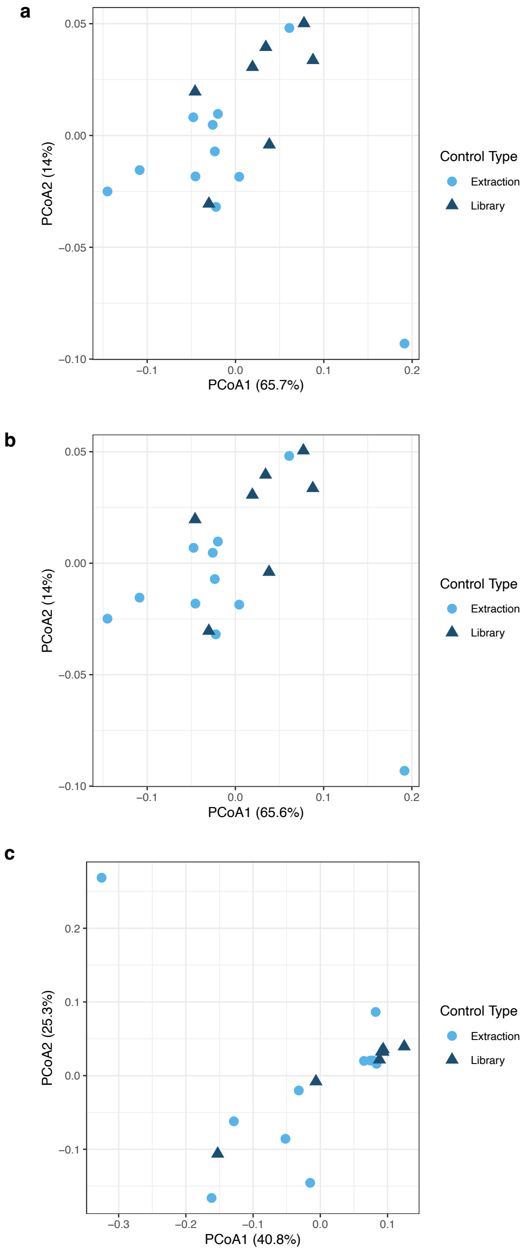

Supplementary Figure S1. Principal Coordinates Analysis (PCoA) based on Bray-Curtis dissimilarity showing the community structure at genus level of the two types of methodological negative controls (Extraction, Library) across each of the main steps of the Quality Control (QC) in our in-house pipeline. **a)** After trimming of sequences. **b)** After deduplication of FASTQs. **c)** After removal of human sequences.

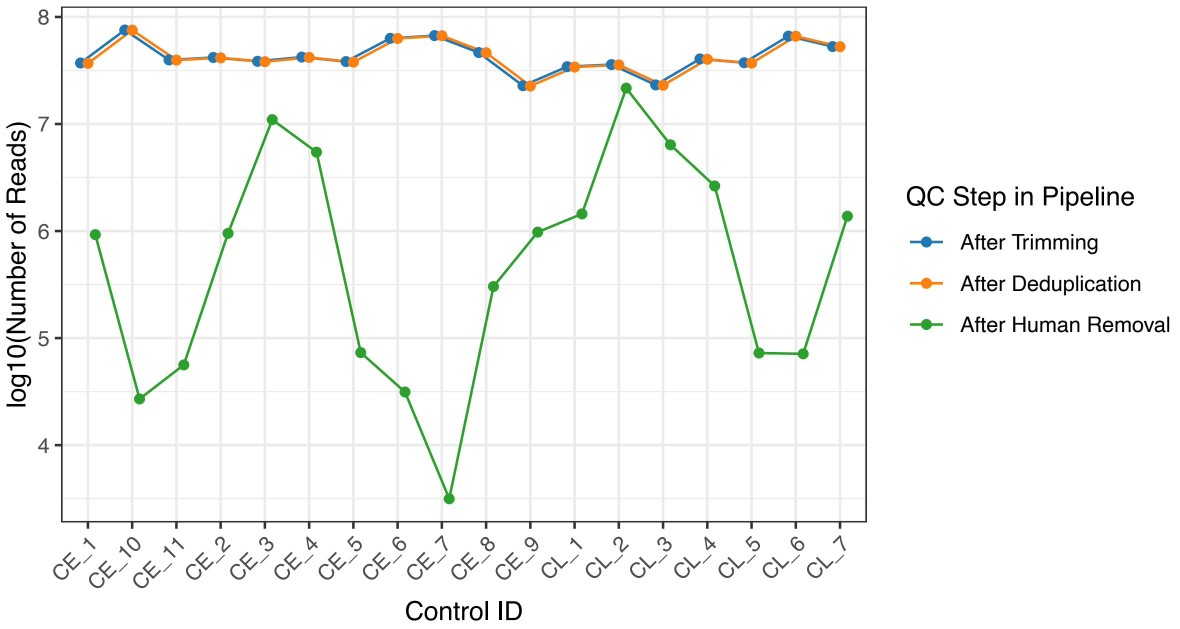

Supplementary Figure S2. Read counts across pipeline QC steps for control samples. The plot shows log-transformed read counts after trimming, deduplication, and human read removal across the different controls. Each line represents a QC step in the metagenomic in-house pipeline

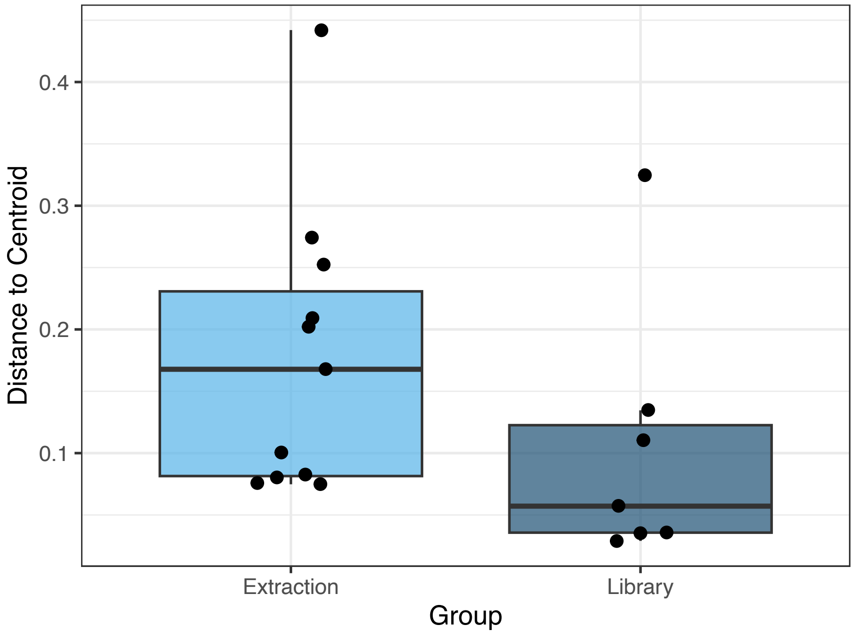

Supplementary Figure S3. Beta Dispersion Analysis comparing the variability in community composition between methodological extraction and library controls. Each point represents a sample's distance to the group centroid based on the Bray-Curtis dissimilarity.

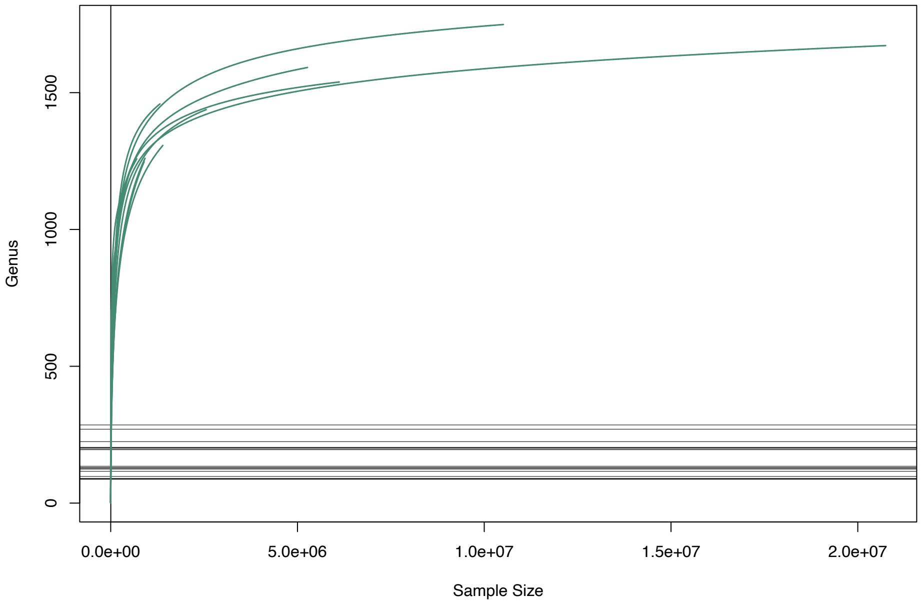

Supplementary Figure S4. Saturation plot for methodological controls.

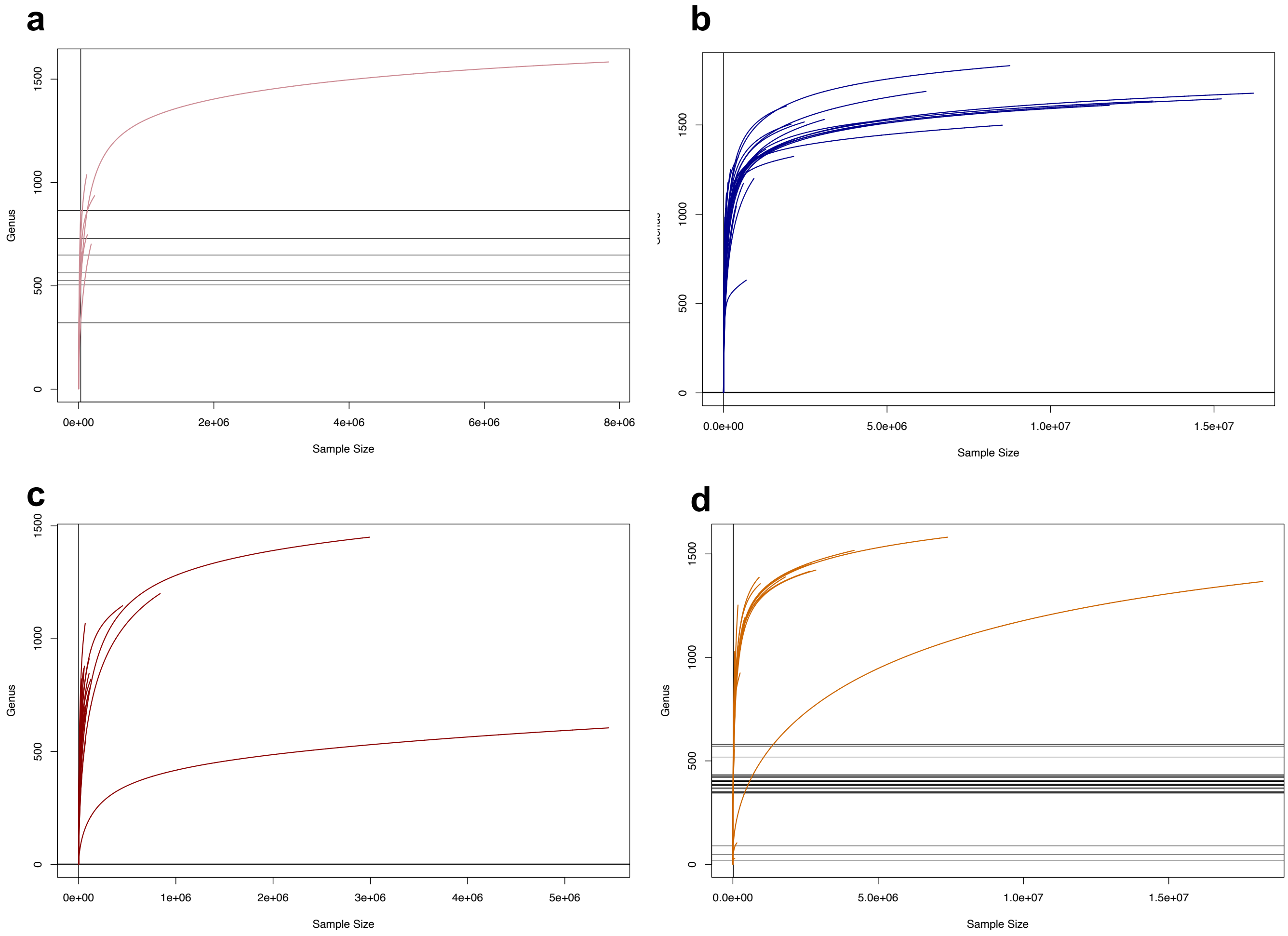

Supplementary Figure S5. Saturation plot for low-biomass samples. A) Amniotic fluid samples. b) Cerebrospinal fluid samples. c) Plasma samples. d) Serum samples.

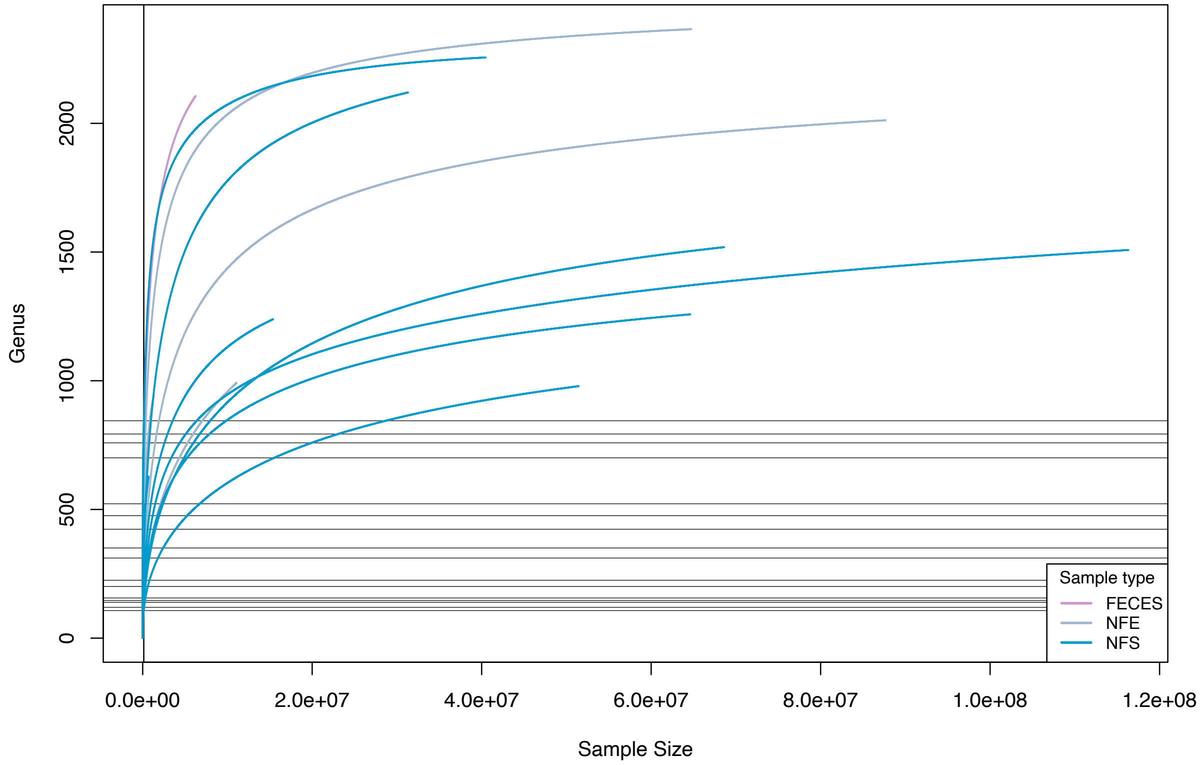

Supplementary Figure S6. Saturation plot for high biomass samples: the feces sample, nasopharyngeal swab samples, nasopharyngeal exudate samples.

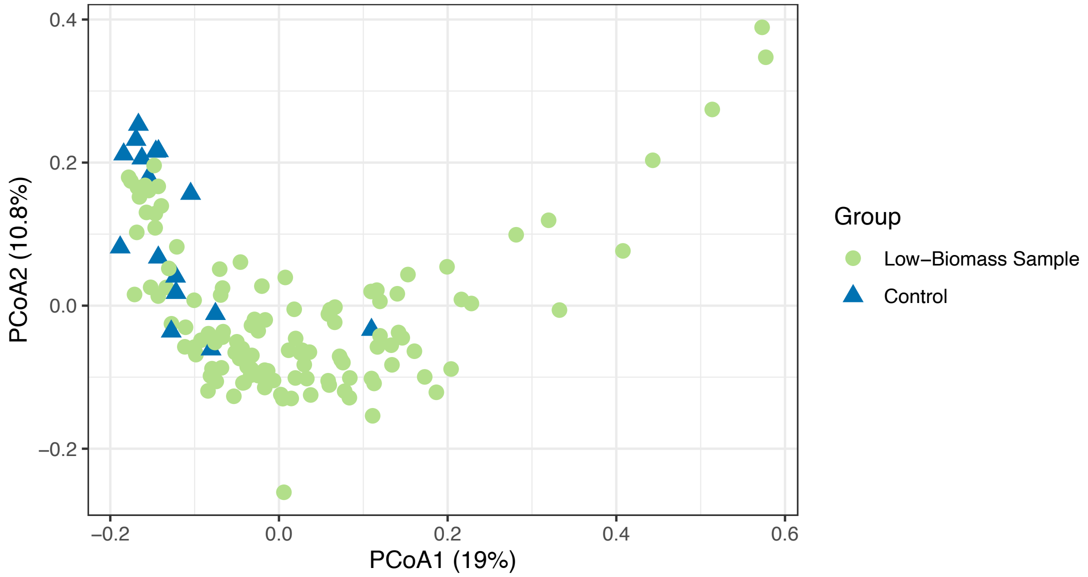

Supplementary Figure S7. Principal Coordinates Analysis (PCoA) based on Bray-Curtis dissimilarity showing the community structure at genus level of low-biomass samples vs controls after read subtraction and the use of decontam as the decontamination strategy.

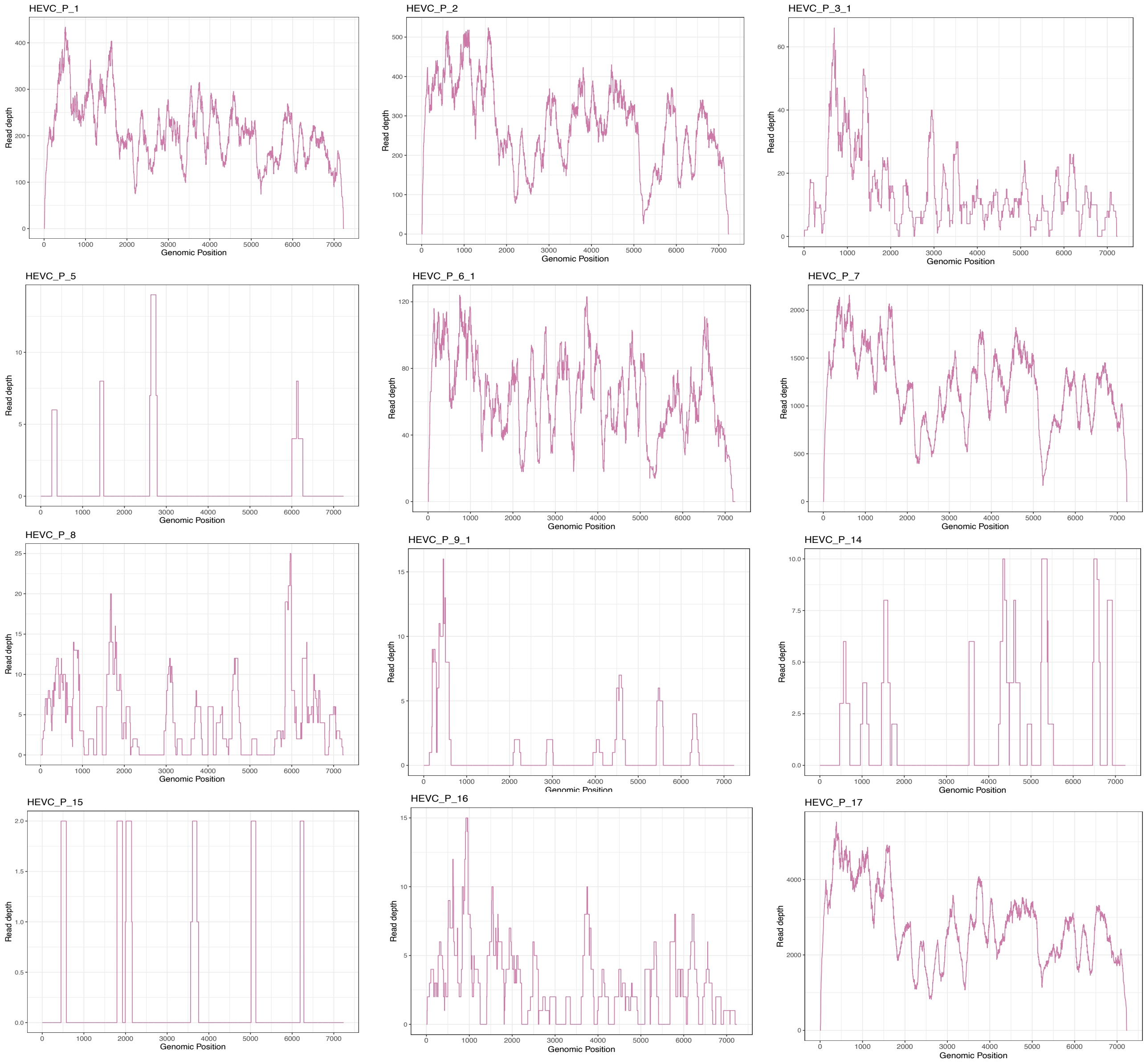

Supplementary Figure S8. HEV genome coverage by sample (HEVC-P-1, HEVC-P-2, HEVC-P-3, HEVC-P-5, HEVC-P-6, HEVC-P-7, HEVC-P-8, HEVC-P-9, HEVC-P-14, HEVC-P-15, HEVC-P-15, HEVC-P-16, HEVC-P-17)**.** Read depth (y-axis) is shown per sable and is not normalized across plots, reflecting each sample's actual read depth.

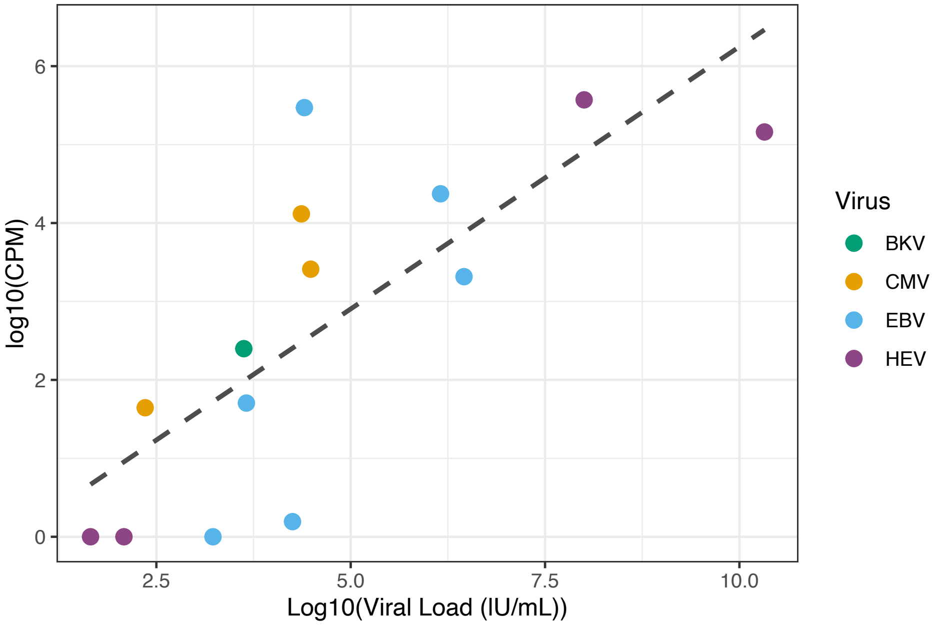

Supplementary Figure S9. Viral Load vs metagenomic detection (CPM). Scatterplot showing log-transformed viral load (IU/mL) and its corresponding log-transformed viral counts per million.

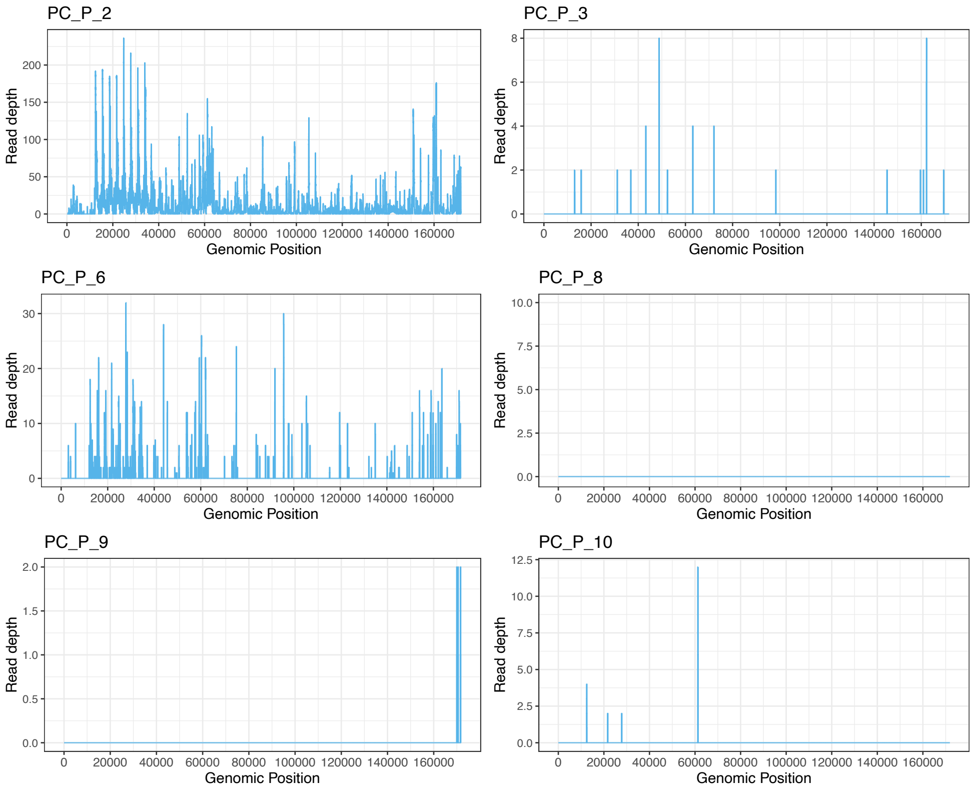

Supplementary Figure S10. EBV genome coverage by sample (PC-P-2, PC-P-3, PC-P-6, PC-P-8, PC-P-9, PC-P-10). Read depth (y-axis) is shown per table and is not normalized across plots, reflecting each sample's actual read depth.

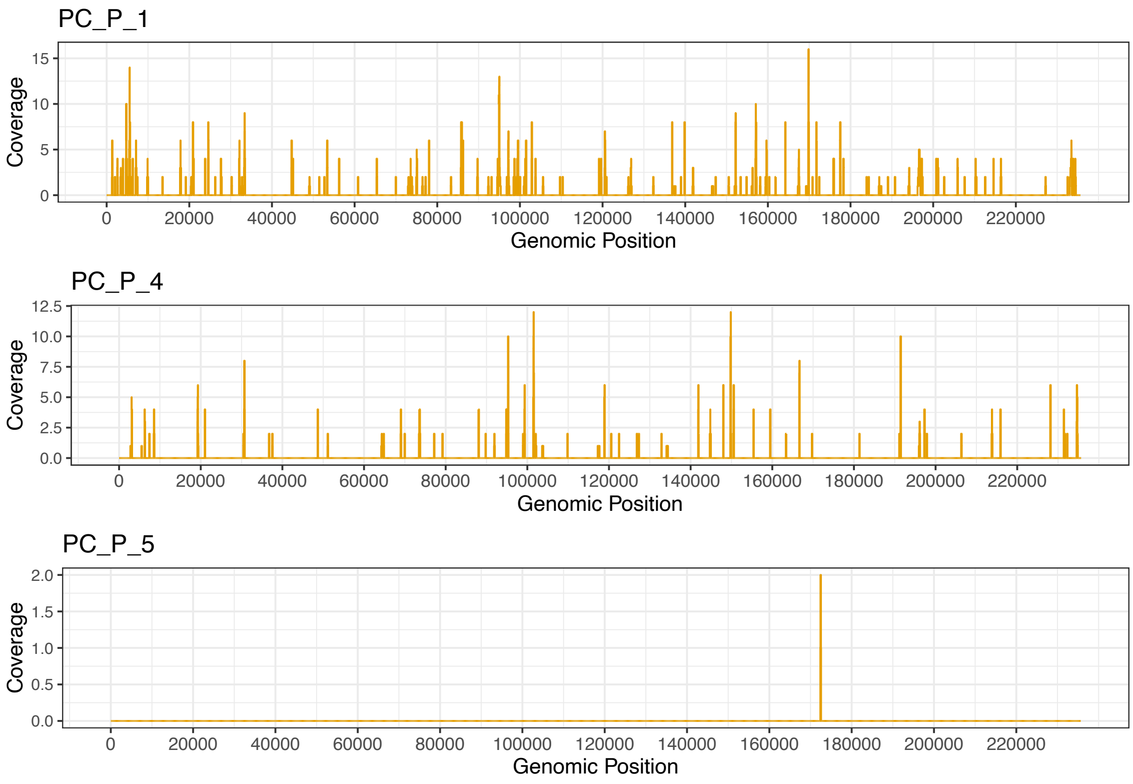

Supplementary Figure S11. **CMV genome coverage by sample (PC-P-1, PC-P-4, PC-P-5)**. Read depth (y-axis) is shown per sample and is not normalized across plots, reflecting each sample's actual read depth.

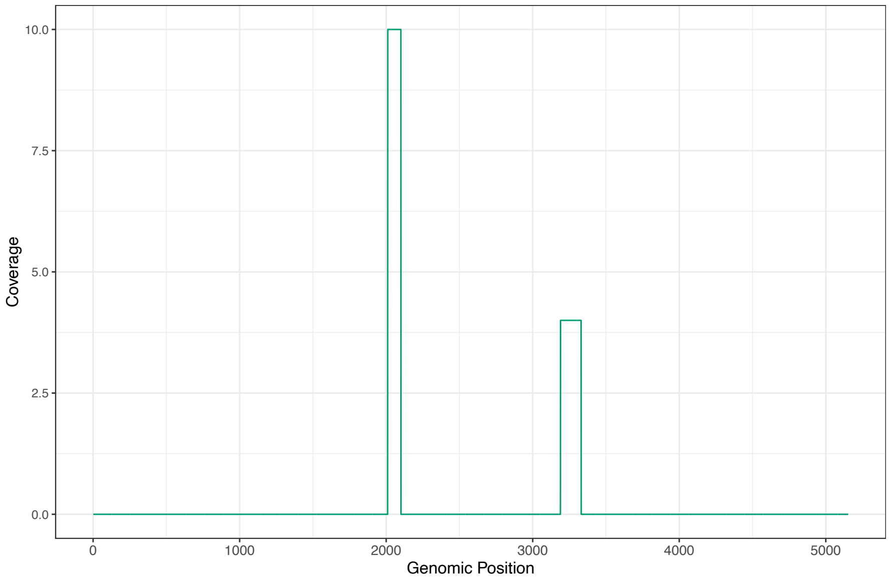

Supplementary Figure S12. BKV genome coverage in sample PC-P-7.

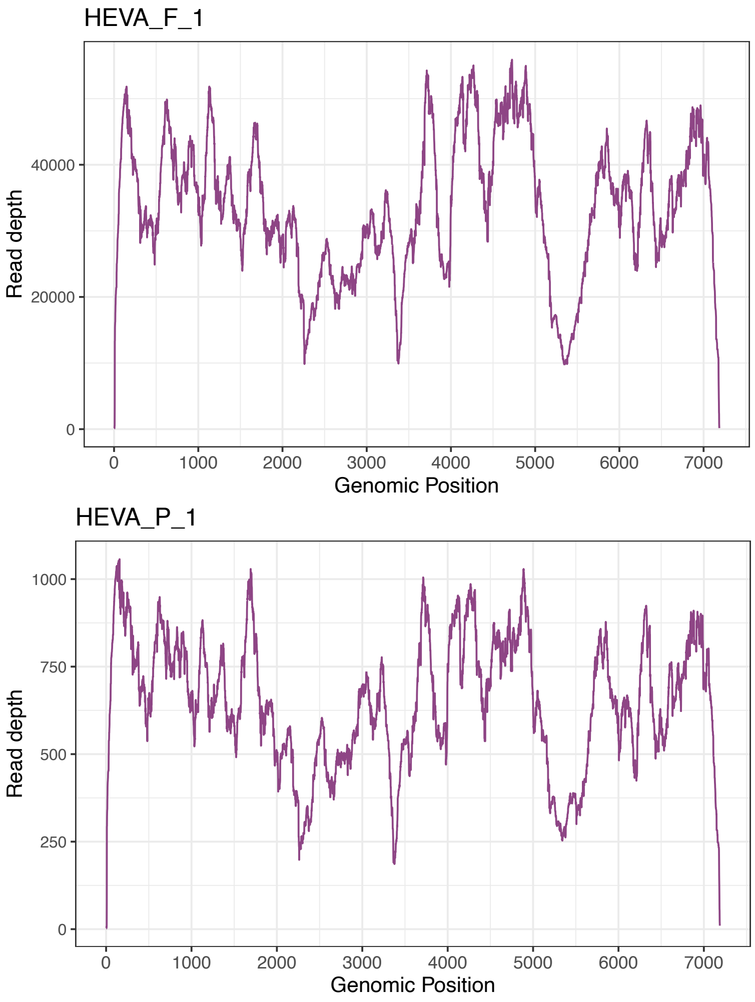

Supplementary Figure S13. HEV-Genotype 1 genome coverage in HEV acute infection samples (HEVA-F-1, HEVA-P-1)**.**

2 Supplementary Tables

Supplementary Table S1. Contaminants (family level) found overlaying our genera from methodological negative controls. These contaminants were prevalent in at least half of all methodological negative controls and had a counts per million (CPM) > 0.5. The table includes family name, NCBI Taxonomy ID, and abundance statistics: prevalence (%), mean counts per million (CPM), median CPM and interquartile range (IQR) of CPM across methodological blank controls.

The complete dataset for Supplementary Table S1 is found in the attached file named Table_S1.csv

Supplementary Table S2. Contaminants (genus level) found overlaying our genera from methodological negative controls. These contaminants were prevalent in at least half of all methodological negative controls and had a counts per million (CPM) > 0.5. The table includes genus name, NCBI Taxonomy ID, and abundance statistics: prevalence (%), mean counts per million (CPM), median CPM and interquartile range (IQR) of CPM across methodological blank controls.

The complete dataset for Supplementary Table S2 is found in the attached file named Table_S2.csv

Supplementary Table S3. Contaminants (genus level) from our contaminant watchlist identified in previous studies. These contaminants were prevalent in at least half of all methodological negative controls and had a counts per million (CPM) > 0.5. The table includes genus name, NCBI Taxonomy ID, and abundance statistics: prevalence (%), mean counts per million (CPM), median CPM and interquartile range (IQR) of CPM across methodological blank controls, in addition to the reference for the study where it was identified.

The complete dataset for Supplementary Table S3 is found in the attached file named Table_S3.csv

Supplementary Table S4. Library-specific contaminants (genus level). Mean and standard deviation of taxon abundance (CPM) across library-specific controls. These taxa were only detected in negative methodological controls included when starting the sequencing library.

Genus	MEAN CPM	Standard deviation of CPM
Candidatus Haliotispira	0.11	0.26
Adlercreutzia	0.20	0.35
Syntrophothermus	0.13	0.26
Anaerotignum	0.12	0.26
Porifericola	0.15	0.35
Mageeibacillus	0.04	0.07
Caminicella	0.28	0.53
Nakaseomyces	8.97	20.93
Aokuangvirus	4.00	10.39
Halothece	2.08	5.17

Supplementary Table S5. Extraction-specific contaminants (genus level). Mean and standard deviation of taxon abundance (CPM) across extraction-specific controls. These taxa were only detected in negative methodological controls included when staring the RNA/DNA extraction procedure.

Genus	MEAN CPM	Standard deviation of CPM
Solilutibacter	0.41	0.99
Oleiphilus	2.68	7.88
Salinivirga	3.46	11.13
Pirellula	11.7	38.43
Candidatus Neptunochlamydia	1.05	2.03
Geovibrio	0.21	0.43
Atopobium	0.84	2.46
Fervidibacillus	14.62	48.04
Ruminiclostridium	0.11	0.32
Peptoclostridium	0.38	0.71
Simiaoa	5.07	16.46
Halothermothrix	0.11	0.32
Acholeplasma	0.72	1.3
Tetrapisispora	7.34	14.34
Trypanosoma	5.06	15.81
Salinirubellus	3.51	11.12
Crenobacter	3.75	11.1
Ferrigenium	32.19	95.13
Sulfuricella	37.22	65.03
Frischella	14.5	44.36
Barnesiella	34.62	94.56
Megalodesulfovibrio	7.31	14.77
Desulfonema	17.50	47.1
Candidatus Electrothrix	3.46	11.13
Candidatus Xiphinematobacter	12.96	28.86
Isosphaera	83.3	252.59
Oerskovia	3.48	11.13
Candidatus Arthromitus	3.41	11.15
Ammonifex	13.64	44.58
Fundicoccus	10.15	33.46
Solobacterium	28.88	70.2
Carboxydochorda	13.54	30.31
Mesomycoplasma	3.57	11.11
Truepera	27.19	65.82
Cryptosporidium	16.25	36.17
Methanofervidicoccus	6.82	22.29
Vectrevirus	7.88	14.21
Polystyrenella	6.53	13.52
Thermoclostridium	3.25	10.73
Ectothiorhodosinus	0.19	0.42
Bisgaardia	0.10	0.32

Basilea	33.36	94.59
Wenyingzhuangia	2.23	7.07
Arenibacter	0.13	0.32
Chondrinema	2.43	6.78
Rubellicoccus	0.2	0.42
Desulfurobacterium	0.14	0.33
Hathewaya	4.01	12.93
Tepidanaerobacter	2.05	6.45
Koinonema	2.07	6.44
Tequatrovirus	1.43	4.11
Otariodibacter	0.09	0.28
Amniculibacterium	0.45	1.07
Cellulophaga	6.42	19.01
Paludibacter	3.1	10.17
Coprobacter	3.00	9.94
Desulfocurvibacter	3.76	6.57
Thermodesulfobacterium	8.59	28.37
Nitratiruptor	0.02	0.06
Sediminispirochaeta	60.09	199.25
Thiospirochaeta	0.02	0.06
Alloacidobacterium	0.02	0.06
Mesoterricola	0.29	0.92
Elusimicrobium	7.04	19.14
Endomicrobium	4.73	12.39
Aurantimicrobium	13.8	38.35
Antiquaquibacter	0.07	0.17
Humibacter	1.56	4.14
Skermania	0.25	0.64
Terribacillus	2.17	6.94
Mesobacillus	1.56	4.95
Proteiniborus	0.67	2.15
Qiania	0.65	1.98
Aminipila	6.84	15.2
Trichormus	8.73	28.83
Cylindrospermum	17.4	57.69
Halococcus	0.05	0.12
Methanosarcina	0.02	0.06
Ceetrepovirus	8.71	28.81
Nitrosophilus	2.36	7.7
Petroclostridium	2.94	9.22
Leptodesmis	0.30	0.92
Kluyveromyces	1.29	4.11
Halorientalis	1.26	4.12
Candidatus Regiella	8.98	15.86
Pontimonas	1.34	4.10
Gloeocapsa	293.42	853.33
Thiolapillus	5.89	19.2
Mangrovivirga	6.79	15.31

Halosquirtibacter	4.95	11.2
Miniphocaeibacter	30.13	95.26
Marinospirillum	0.49	1.12
Candidatus Endoriftia	0.67	1.51
Aestuariibaculum	2.20	5.00
Pelobacter	33.24	98.83
Actinobaculum	10.45	32.66
Chryseomicrobium	11.77	32.14
Murdochiella	7.37	23.36
Aminomonas	0.88	2.11

Supplementary Table S6. Percentage of reads belonging to contaminants identified in the watchlist according to type of clinical sample. Mean, standard deviation (SD), and median percentage of contaminant reads are shown for each sample type.

Sample type	Number of samples	Mean percentage of contaminant reads (%)	SD	Median (%)
**Amniotic fluid**	7	98.6	1.34	98.9
**Serum**	24	97.7	7.33	99.86
**Nasopharyngeal exudate**	4	97.3	2.47	97.85
**Cerebrospinal fluid**	68	96.9	13.3	99.41
**Plasma**	36	94.4	16.37	98.22
**Nasopharyngeal swab**	11	92.6	16.8	99.6
**Feces**	1	48.9	NA	NA

Supplementary Table S7. Table of counts per million (CPM) of genus contaminants identified in the watchlist across all clinical samples. Rows correspond to genera included in the defined contaminant watchlist at genus level. Columns correspond to individual clinical samples (sample IDs). Cell values represent the proportion of non-human reads assigned to each genus expressed as CPM.

The complete dataset for Supplementary Table S7 is found in the attached file named Table_S7.csv

Supplementary Table S8. Summary of metagenomic sequencing and assembly results for all plasma samples belonging to a chronic infection from Hepatitis E Virus (HEV). The table shows each sample with each corresponding viral load (IU/mL), the normalized read counts (counts per million, CPM), the percentage of genome covered, number of corresponding contigs assembled, N50 statistics, longest contig assembled and total length of all contigs.

Sample	Viral Load (IU/mL)	CPM	Genome Covered (%)	Number of contigs	N50	Longest contig	Total length
**HEVC_P_1**	7,00E x 10^5	375149.13	99.71	1	7195	7195	7195
**HEVC_P_2**	8,10 x 10^5	366180.11	99.72	1	7217	7217	7217
**HEVC_P_3**	1,00 x 10^5	19407.01	96.65	3	3068	3068	4975
**HEVC_P_4**	3,50 x 10^1	0.00	0.00	0	NA	NA	NA
**HEVC_P_5**	1,00 x 10^3	457.63	9.11	0	NA	NA	NA
**HEVC_P_6**	5,20 x 10^4	173166.00	99.12	1	7174	7174	7174
**HEVC_P_7**	2,70 x 10^6	507993.97	99.73	1	7294	7294	7294
**HEVC_P_8**	4,20 x 10^4	10148.54	70.05	5	579	988	2788
**HEVC_P_9**	1,50 x 10^3	746.33	24.26	1	376	376	376
**HEVC_P_10**	1,10 x 10^2	45.06	2.55	0	NA	NA	NA
**HEVC_P_11**	9,90 x 10^1	167.11	2.07	0	NA	NA	NA
**HEVC_P_12**	3,00 x 10^2	0.00	0.00	0	NA	NA	NA
**HEVC_P_13**	1,10 x 10^3	82.09	3.41	0	NA	NA	NA
**HEVC_P_14**	2,50 x 10^3	1533.31	29.18	0	NA	NA	NA
**HEVC_P_15**	4,80 x 10^3	640.61	11.35	0	NA	NA	NA
**HEVC_P_16**	2,80 x 10^4	19021.05	75.40	6	284	430	1780
**HEVC_P_17**	2,20 x 10^6	709863.70	99.77	11	1265	2714	10406

Supplementary Table S9. Table summarizing sequencing and assembly metrics for multiple respiratory virus coinfections across clinical samples. For each virus detected by qPCR, the corresponding Ct value, counts per million (CPM), and assembly statistics are reported, including the number of contigs, N50, longest contig length, total assembled length, and the number of reads mapped. Lower Ct values generally correspond to higher CPMs and more complete genome assemblies, as reflected by larger contig lengths and total assembled bases. Abbreviations: MPV, Human metapneumovirus; ADV (VR), adenovirus; BoV, bocavirus; CoVOC43; human coronavirus OC43; HPIV-3, human respirovirus 3; EV, enterovirus; RV, rhinovirus.

Sample ID	Tested viruses	Ct	CPM	Contigs	N50	Longest contig	Total length
**PC_NFS_1**	MPV	34.00	0.38	2	377	377	600
	ADV (VR)	26.67	1.11	1	310	310	310
**PC_NFS_2**	ADV (VR)	34.13	0.00	0	NA	NA	NA
	BoV	31.88	0.00	0	NA	NA	NA
	HIPV-3	36.29	0.91	0	NA	NA	NA
	RV	30.71	2.41	1	342	342	342
**PC_NFS_3**	BoV	19.00	438.11	1	5477	5477	5477
	HPIV-3	24.49	3892.11	1	15427	15427	15427
**PC_NFS_4**	MPV	32.96	0.00	0	NA	NA	NA
	HPIV-3	24.13	69440.38	12	7367	7367	13415
	RV	31.8	0.00	0	NA	NA	NA
**PC_NFS_5**	ADV (VR)	25.35	76.43	32	842	2909	24095
	RV	25.18	1666.75	1	7328	7328	7328
**PC_NFS_6**	HPIV-3	23.95	2843.46	1	15430	15430	15430
	RV	23.52	2190.41	1	7132	7132	7132
**PC_NFS_7**	HPIV-3	33.49	7486.83	12	482	627	5315
	RV	28.55	21860.49	1	7090	7090	7090
**PC_NFS_8**	EV	30.74	26.22	3	3920	3920	5758
	BoV	31.04	0.00	0	NA	NA	NA
	HPIV-3	36.74	16.83	1	388	388	388
	RV	27.73	76.71	1	7055	7055	7055
**PC_NFS_9**	MPV	36.13	59.12	0	NA	NA	NA
	HPIV-3	23.49	735609.42	1	15440	15440	15440
	RV	28.15	3990.54	1	7096	7096	7096
**PC_NFS_10**	HPIV-3	24.74	781.56	0	NA	NA	NA
	RV	31.37	0.00	0	NA	NA	NA
**PC_NFS_11**	EV	30.71	14.50	3	1235	2135	3739
	ADV (RV)	21.67	269.22	18	3324	9350	3568
	BoV	32.3	0.00	0	NA	NA	NA
	RV	30.14	22.02	1	6588	6588	6588
**PC_NFE_1**	CoV OC43	26.29	10.15	0	NA	NA	NA
	HPIV-3	16.15	379028.90	27	13999	26610	13999

**PC_NFE_2**	HPIV-3	28.21	99.71	1	15392	15392	15392
	RV	21.38	1486.00	2	7163	7163	7534
**PC_NFE_3**	HPIV-3	25.72	458.01	1	15424	15424	15424
	RV	30.58	1.43	1	7066	7066	7066
**PC_NFE_4**	HPIV-3	23.85	316274.85	1	15440	15440	15440
	RV	32.76	53.66	1	1577	1577	1577
